# A Personalized and Smartphone-Based Serious Gaming App Targeting Cognitive Impairments in Alcohol Use Disorder: Double-Blinded, Randomized Controlled Efficacy Trial Among Outpatients

**DOI:** 10.2196/67167

**Published:** 2025-10-07

**Authors:** Nicolaj Mistarz, Laust Vind Knudsen, Anna Mejldal, Kjeld Andersen, Anneke Goudriaan, Lotte Skøt, Tanja Maria Michel, Angelina Isabella Mellentin

**Affiliations:** 1 Research Unit of Psychiatry Department of Clinical Research University of Southern Denmark Odense C Denmark; 2 Amsterdam University Medical Centre Department of Psychiatry University of Amsterdam Amsterdam The Netherlands; 3 Amsterdam Institute for Addiction Research Department of Research Arkin Amsterdam The Netherlands; 4 Brain Research-Inter-Disciplinary Guided Excellence Department of Clinical Research University of Southern Denmark Odense C Denmark

**Keywords:** alcohol use disorder, cognitive impairment, cognitive training, serious gaming, pilot study, feasibility study, cognitive training, smartphone, apps, monitoring, gamified, randomized controlled trial

## Abstract

**Background:**

Alcohol use disorder (AUD) is associated with cognitive impairments that are known to affect the outcomes of conventional treatment. Digital cognitive training programs have been examined as a possible way of addressing these overlooked challenges. Existing findings regarding the efficacy of such training programs are divergent, and further studies are warranted to examine more engaging cognitive training programs using the latest technology. Smartphone-based training built upon the principles of serious gaming would not only increase the accessibility of the program, but it could also increase the motivation of the patients, potentially maximizing adherence to the training program.

**Objective:**

The aim of the present feasibility and efficacy study was to examine the feasibility and acceptability of the Brain+ Alco-Recover app (Brain+ A/S) with gamified elements among patients with AUD when delivered as an add-on to treatment-as-usual (TAU) and with minimal guidance from health care practitioners. In addition, the effects on cognitive and alcohol-related outcomes were examined.

**Methods:**

A total of 72 outpatients were randomized into either group A, experimental + TAU (n=36), or group B, sham + TAU (n=36), and they had to complete a 1-month training program in addition to primary treatment. Self-reported experience at the 6-month follow-up as well as actual game usage was used to determine the feasibility of the training program. Cognitive performance and alcohol consumption were assessed as well.

**Results:**

The patients in both groups reported a high level of acceptability, and up to 83% of the patients in the experimental group met the minimum requirements for the usage of the app. The experimental group also demonstrated significant improvements in working memory (*P<*.001). Although no significant differences were found between the 2 groups regarding clinical outcomes, a greater reduction in alcohol consumption was evident at the 6-month follow-up in the experimental group.

**Conclusions:**

The acceptability and adherence to the minimum training requirements deems the gamified Brain+ app as a feasible tool for cognitive training when delivered as an add-on to TAU. Furthermore, the potential improvements in cognitive functions should be further replicated in a larger-scale trial to assess whether these could be used to improve the treatment of AUD in the future.

**International Registered Report Identifier (IRRID):**

RR2-10.3389/fpsyt.2021.727001

## Introduction

Individuals who have experienced long-term exposure to the neurotoxic effects of alcohol may show signs of cognitive impairment [1,2]. Patients with alcohol use disorder (AUD) experience partial recovery after successfully completing alcohol treatment and sustaining abstinence, but recent findings also suggest that some cognitive deficits persist in the domains for processing speed and attention (PSA), working memory (WM), and executive functions (EF), and impairments in learning and memory may persist during periods with abstinence [3,4]. Even after a year of abstinence, patients with AUD still display a broad range of diffuse dysfunctions in controlled and effortful processes such as EF and WM [1,5,6].

Unimpaired effortful cognitive processes are important in the patients’ everyday lives, but also in aiding them to exert control over their own impulses toward alcohol-related cues, which in turn could increase the likelihood of abstaining from alcohol [7]. This is corroborated by recent findings, where patients with AUD who had a lower score on the Montreal Cognitive Assessment compared to healthy individuals had an increased risk of relapse at 3 and 6 months after discharge [8]. In addition, impaired attention and task shifting by means of performance on the Trail-Making Test-B was associated with a higher number of drinking days at the 3- and 6-month follow-up. Systematic reports on AUD and other substance use disorder (SUD) have also found medium effect sizes for associations between general cognition and treatment adherence for cognitive behavioral therapy, as well as impaired decision making and increased risk of relapse [9,10]. Although these findings were heterogeneous and based on various populations with SUD, the negative impact on treatment outcomes underlines the importance of recovering a broad range of cognitive functions among patients with AUD.

Therapeutic approaches focusing on restoring cognitive functions often consist of recurring, behavioral tasks that, if maintained for a longer period, result in neuroplastic changes, which in turn may be exhibited as improvements in daily activities [11]. The effects of cognitive training can be assessed according to how closely the assessment resembles the training task. Increased performance in cognitive domains targeted by similar to those prespecified in the cognitive training program is indicative of a so-called near-transfer effect [12]. On the other hand, improved performance in domains that are not related to the domains actively targeted by the training program is known as far-transfer effects [12].

The evidence of near-transfer effects among patients with AUD has been demonstrated using a wide range of different digital and computerized cognitive training programs [2]. These interventions have been shown to be effective in improving different cognitive domains, but especially the domains for EF and WM have been scrutinized [13]. Although evidence suggests that cognitive training designed to target specific domains can provide near-transfer effects [14], an improvement in one domain may not optimally address the diffuse and heterogeneous cognitive deficits that are evident in patients with AUD. In keeping with this notion, only preliminary findings are evident demonstrating far-transfer effects of cognitive training among patients with AUD [13]. This suggests that cognitive training programs focusing on a single domain, or a few closely related domains, may be an inadequate strategy for targeting the wide range of cognitive impairments exhibited by patients with AUD. More complex programs consisting of a variety of training tasks, each targeting a set of specific cognitive processes, could thus be needed. These types of multidomain, cognitive training programs have been shown to be feasible as well as capable of improving performance on a variety of cognitive tasks among patients with AUD [15-18]. Although the studies were mainly centered around the effects on higher-order cognitive functions such as WM and problem solving, these findings suggest that using more multifaceted programs may be an effective method for improving a broader range of cognitive processes, which in turn could be beneficial for the abilities required for daily activities.

In the past decade, there has been a growing interest in uncovering whether the effects of cognitive training can be transferred to clinical outcomes [2]. So far, evidence suggesting that cognitive training can improve alcohol-related outcomes has been scarce [19]. A few studies on domain-focused cognitive training [20,21] have examined the effects on alcohol consumption, but no significant reductions were found when compared to the control groups. Contrasting the findings from domain-focused programs, 3 small efficacy randomized controlled trials used training programs targeting multiple cognitive domains [18,22,23], and 2 of these reported improved abstinence rates among patients [18,23]. Although these findings were observed in only 2 studies, the results suggest that interventions addressing a broader range of cognitive functions may hold promise for improving clinical outcomes.

One possible factor that might explain the diverging findings regarding clinical outcomes could be the level of motivation [24]. The patient’s motivation to complete a training program can be optimized if the tasks are kept adequately challenging while not being too overwhelming [25]. Earlier versions of computerized cognitive training have relied on less engaging paradigms based on an experimental setting, where the patients were required to use proprietary software that could only be used on a stationary computer [22]. Not only did this limit the accessibility, but it would also mean that training days would need to be scheduled regularly with a researcher or clinician. Early versions of goal management training and software targeting WM were also characterized by simple user interfaces with a minimum number of visual elements [5]. The lack of visually capturing features were outweighed by more text-based tasks that were closer in resemblance to standardized neuropsychological assessment batteries [17,22], and this could make the training at risk for being perceived as trivial and decreasing the treatment adherence [5]. Nonetheless, advancements in mobile health (mHealth) technology have made it easier to create a personalized experience and more visually engaging training programs that incorporate gamification [26] and serious gaming elements [27]. Contrasting previous training programs, the latest technological capabilities would also allow outpatients admitted to conventional treatment to access the training program from home, thus reducing the required visits to a treatment facility [27,28].

The aims of this feasibility and efficacy study were to assess the acceptability and feasibility of a semiguided, smartphone-based, serious gaming app administered as an add-on to conventional treatment among patients with AUD. As secondary objectives, we wanted to explore whether the training program could show improvements in cognitive performance and if potential benefits on alcohol consumption and craving could be observed.

## Methods

### Ethical Considerations

The present study was approved by the Research Ethics Committee for the Region of Southern Denmark (S-20200199), and it was conducted in accordance with the Declaration of Helsinki [29]. This study was not registered in a public trial registry, but a protocol for the trial was published prior to the inclusion of patients [30], and registered under the International Registered Report Identifier (IRRID; RR2-10.3389). No amendments were made to the original protocol, but minor changes to the total sample size and age criteria were incorporated into the study (see Multimedia Appendix 1). Further, the reporting of this study is in accordance with the Consolidated Standards of Reporting Trials (CONSORT) 2010 Statement: updated guidelines for reporting parallel randomized trials [31] and the CONSORT-EHEALTH (Consolidated Standards of Reporting Trials of Electronic and Mobile Health Applications and Online Telehealth) checklist (see Multimedia Appendix 2 [32]).

There were no known harmful effects of the smartphone-based training program. As all patients in this study were offered either pharmacological or psychological treatment, the study was not evaluated as posing any ethical challenges. Finally, all patients were informed that they could withdraw their consent without it having any implications for their current or future treatment options. If the patients chose to withdraw their consent, all their data would be erased.

### Trial Design

This pilot trial was conducted as a double-blinded, randomized controlled trial with 2 parallel groups. An urn randomization technique was used without any stratification [33], and the allocation of patients to the groups was performed by an independent statistician.

### Recruitment

Patients from the publicly financed, outpatient alcohol treatment clinic in Odense, Denmark, were consecutively invited to take part in the study until a sample of 72 patients was obtained. The recruitment took place between September 2021 and September 2022. All patients attending the clinic in Odense undergo a 3-month treatment program consisting of either pharmacotherapy or psychotherapy or a combination of both, where the latter is based on manualized combined motivational interviewing and cognitive behavioral therapy [34]. During the primary treatment, a health care professional conducted a clinical assessment of the patients according to the *International Classification of Diseases, Tenth Revision* *(ICD-10)* [35]. The Addiction Severity Index (ASI) was used to assess sociodemographic characteristics to generate an addiction severity profile for each patient [36].

### Eligibility Criteria

Patients were eligible to participate in the study if they: (1) had a confirmed diagnosis of AUD (ie, harmful use or abuse or dependence); (2) had access to a smartphone or tablet that met the requirements for using the gamified cognitive training app; (3) were between 18 and 70 years of age; (4) spoke Danish; (5) provided informed consent to participate; and (6) had completed detoxification prior to study inclusion if deemed necessary. Patients were excluded if they had a diagnosis of SUD other than AUD, a severe mental disorder with suicidal, manic or psychotic symptoms, a neurological disorder, or terminal illness.

### Procedure

Eligible patients were informed about the study by their health care providers. Patients who expressed interest in participating were referred to a research assistant who provided verbal and written information about the study following a standardized procedure. The patients signed an informed consent form, and they were further screened for eligibility and randomly allocated to an experimental or sham training group. Following this, a pretreatment assessment of cognitive and clinical outcomes was conducted, and lastly the research assistant helped the patients with installing the app on their smartphone or tablet.

#### Allocation and Blinding

Eligible patients were consecutively randomized to one of 2 groups: (A) an experimental version of the cognitive training program + treatment as usual (TAU) (n=36), or (B) a sham version of the cognitive training program + TAU (n=36). The patients were blinded to their allocated group, and the research assistants who performed the randomization were also unaware of which version of the training app they helped to install on the patients’ smartphones or tablets. This was achieved by assigning a random number to the 2 versions of the training app, and these 2 designated numbers were only known to an independent researcher who did not take part in the assessment of the patients.

#### Assessment and Outcomes

Sociodemographic and clinical data were extracted from the outpatient clinic’s clinical database containing data from the initial clinical assessments performed by the health care professionals. Assessments were conducted by research assistants at study enrollment (T0), posttreatment (T1), and at 6-month follow-up (T2).

#### Acceptability and Feasibility Outcomes

The acceptability of the Brain+ Alco-Recover (Brain+ A/S) app was determined by means of the System Usability Scale (SUS) and was only administered at T2. This scale evaluates different elements of the user-interface and assesses the confidence of the user in using the training program [37]. The criterion for the feasibility outcome was based on the actual usage of the individual games in the training program. For the experimental group, a minimum requirement of 5 completed game trials for each of the individual game types was predetermined and this was equal to approximately 80 minutes of total training time (ie, 20% of the instructed duration for the predefined training program). It was expected that the adherence to the training program would be much lower than the instructions provided to the patients due to the lack of a notification system and that the patients trained at home. Thus, for the training program to be deemed as feasible, a criterion was set, where at least half of the patients in the experimental group had to meet these minimum requirements for each game type.

#### Cognitive and Clinical Outcomes

Cognitive performance was measured with assessment versions of the various training games in Brain+ Alco-Recover at T0 and T1. The performance scores in 2 cognitive domains were prespecified as PSA (ie, the in-games Perception Speed and Attention Island) and WM (ie, the in-games Memory Lane and Bulky Codes).

Alcohol consumption was measured at T0, T1, and T2 using the following 2 items derived from the alcohol module in the ASI: (1) days with any alcohol consumption in the past 30 days, and (2) days with excessive drinking (ie, 3 units or more) in the past 30 days [36]. Mean craving level and highest craving level during the past week and the past 30 days were measured at T0, T1, and T2 using the visual analogue scale (VAS) [38].

### Intervention

The cognitive training app, Brain+ Alco-Recover, was adapted from the publicly available Brain+ Recover app [39], which was developed by neuropsychologists at the Centre for Traumatic Brain Injury, University of Copenhagen in collaboration with the company Brain+. To test the effects of the training program with high ecological validity, the Brain+ Alco-Recover app was used by the patients at home without any guidance from the researchers.

#### Experimental and Sham Version of the Cognitive Training Program

The experimental version of Brain+ Alco-Recover consisted of a personalized training program initially created from the preassessment, and the difficulty level was adjusted to fit the patient’s performance level measured during the preassessment. The personalized, daily training sessions consisted of 6 different games (see Multimedia Appendix 1 for detailed information and screenshots of each game), which were combined in 2 blocks of 4 games each. The training blocks alternated between the selection of games, but all the games appeared with the same frequency. The in-game algorithm ensured that the 2 blocks were personalized in terms of the duration of each game, and the cognitive domains in which the patient underperformed during the preassessment were thus targeted for more intensive training. Each game took on average 2 minutes and 40 seconds to complete, but 1 training block lasted 10 minutes. The sham version of the training program was nearly identical to the experimental version, except for the omission of 1 game (see Multimedia Appendix 1 for details). While the games in the sham condition remained adaptive to the patient’s performance, the level of difficulty was fixed to minimize the potential for cognitive improvement (eg, by preventing further reductions in exposure time). Importantly, we identified a discrepancy between the sham and experimental versions of the app that resulted in differences in baseline measurements (Table 1). To ensure this discrepancy did not bias our statistical analysis, we focused on within-group change by analyzing improvement from baseline to final measurement, rather than relying on absolute scores.

Previous studies have found an effect of training sessions lasting 45-60 minutes [16,20], but as we wanted to establish the feasibility of the Brain+ app in its early stages, we chose to incorporate short but frequent training sessions. Patients in the experimental and sham group were instructed to use the app for 20 minutes a day, 5 days a week, for 1 month (ie, a minimum of 400 minutes or 6 hours and 40 minutes of total in-game time for the entire intervention). This resulted in a daily training session that consisted of 2 blocks of 10 minutes each (ie, 2 blocks of 4 games each). As a notification system was not yet developed for the app, patients were reminded to complete their training sessions, when they had to visit the outpatient facility, but they were not guided any further. Thus, this was a semiguided training program in which patients were neither monitored for their actual game progress nor assisted with completing any games, which also meant that there were no implications if a patient failed to adhere to the training program. It was not possible to log the time spent on the training; instead, the total duration was estimated based on the number of completed game trials.

### Statistical Analyses

The baseline characteristics of patients in both study arms were summarized using means and standard deviations (SD) for continuous variables, and frequencies and percentages (%) for categorical variables. Differences between the 2 arms were assessed using 2-tailed, independent samples *t* tests for continuous variables and chi-square tests for categorical variables.

To determine whether 50% of the patients in each group completed the minimum of 5 game trials for each game type, our feasibility was analyzed using Fisher Exact test under the intention-to-treat (ITT) principle, including all randomized participants. In terms of cognitive outcomes, the within-group change from baseline to posttreatment in domain scores (ie, PSA and WM) as well as individual game scores, were analyzed using linear mixed-effects models with random intercepts for participants, fixed effects for time, and a group-by-time interaction. An unstructured covariance matrix and robust standard errors were used. These models inherently account for all available data under the assumption of missing at random and are consistent with the ITT principle. Analyses were also conducted per-protocol (PP), including only participants who completed ≥ 5 game trials for each game type.

Clinical outcomes, which included drinking days (DD), heavy drinking days (HDD), mean and highest craving level (MCL and HCL, respectively), were analyzed using similar linear mixed-effects models across baseline (T0), posttreatment (T1), and 6-month follow-up (T3), with a group-by-time interaction, unstructured covariance, and robust standard errors. These analyses followed the ITT principle. No formal power calculation was conducted; results are interpreted as efficacious. All analyses were performed using Stata 18 (StataCorp LLC). A 2-sided *P* value<.05 was considered statistically significant.

## Results

### Participants

**Figure 1:**
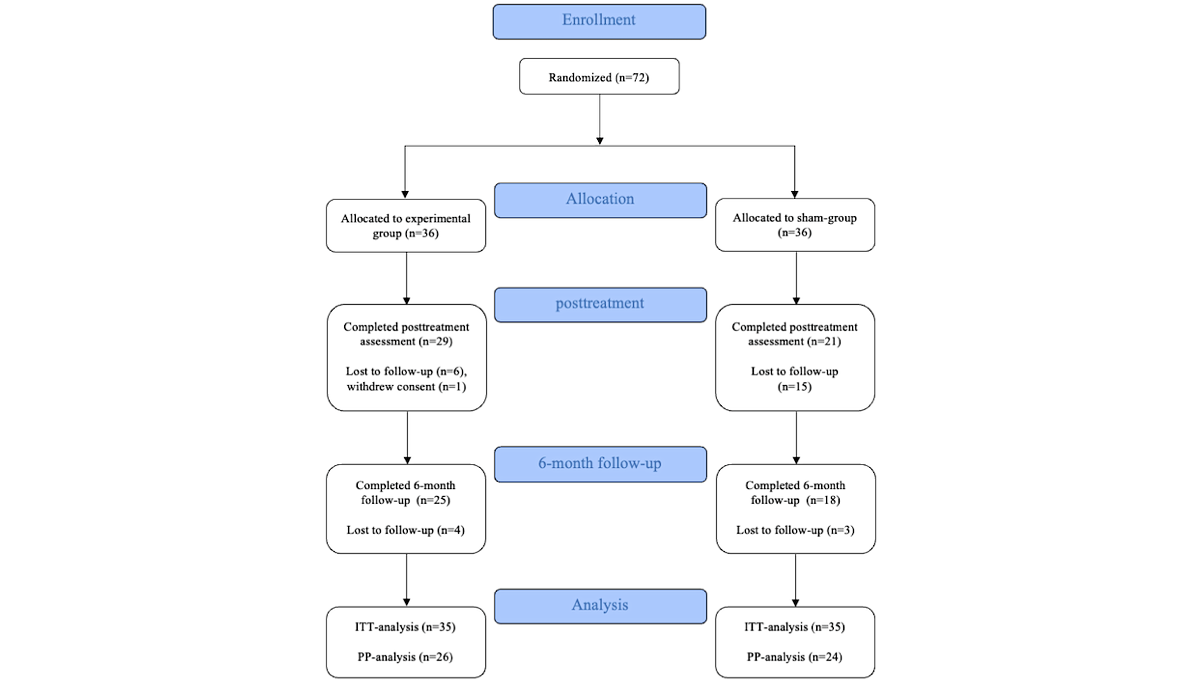
Preferred Reporting Items for Systematic Reviews and Meta-Analyses (PRISMA) chart depicting the flow of participants from allocation to the last follow-up after 6 months. ITT: intention-to-treat; PP: per-protocol.

A total of 72 patients were randomized to either the experimental group (n=36) or the sham group (n=36). One patient in the experimental group withdrew consent to participate after the baseline assessment and thus data for this person were deleted (see Figure 1). Fifty patients completed the posttreatment assessment (ie, experimental group: n=29; sham group: n=21), and 43 patients completed the 6-month follow-up assessment (ie, experimental group: n=25; sham group: n=18). It was not possible to reach any of the missing patients for the posttreatment assessment or at the follow-up after 6 months; thus, no further information on reasons for discontinuing the study could be retrieved.

### Baseline Characteristics

No significant differences were found between the experimental and sham groups regarding age, gender, alcohol consumption, and craving variables (see Table 1). There were 77% (n=27) male patients in the experimental group and 72% (n=26) were male in the sham group. The mean age of the patients in the experimental groups was 45.3 (SD 10.1) years, whereas the patients in the sham group were aged 41.4 (SD 11.5) years on average. The cognitive assessment revealed significant differences between the experimental and sham groups in the domain for PSA as well as the domain for WM (*P*<.001). Inspecting the individual mean domain scores, the experimental group had a higher cognitive performance when compared to the sham group.

**Table 1:** Baseline characteristics of the patients. A higher mean domain/individual game score indicates better cognitive performance.

Baseline					Experimental (n=35)		Sham (n=36)		*P* value
**Sociodemographic variables**									
	Age (years), mean (SD)				45.3 (10.1)		41.4 (11.5)		.14
	Gender, n (%)								.78
			Male	27 (77)		26 (72)			
			Female	8 (23)		9 (25)			
**Clinical variables**									
	Diagnosis, n (%)								
			Harmful use (F10.1)	2 (6)		2 (6)			
			Alcohol dependence (F10.2)	33 (94)		33 (92)			
	Previous treatment for AM^a^, n (%)								
			Yes	23 (66)		22 (61)			
			No	12 (34)		13 (36)			
	Age of onset of HD^b^, mean (SD)				28.3 (10.8)		28.1 (11.0)		.92
	DD^c^ in the past 30 days, mean (SD)				17.9 (10.8)		14.8 (10.6)		.23
	HDD^d^ in the past 30 days, mean (SD)				16.5 (10.6)		13.7 (11.1)		.29
	ACL^e^ in the past 30 days, mean (SD)				3.9 (2.6)		4.1 (2.7)		.72
	HCL^f^ in the past 30 days, mean (SD)				5.4 (3.4)		5.3 (3.4)		.93
**Cognitive variables, mean (** **SD** **)**									
	PSA^g^ domain				440.6 (166.3)		70.4 (70.5)		<.001
	Attention Island				86.3 (71.8)		12.3 (4.3)		<.001
	Speed Perception				650.1 (152.1)		208.6 (134.5)		<.001
	WM^h^ domain				210.3 (36.8)		127.4 (7.7)		<.001
	Bulky Codes				212.3 (39.8)		137.2 (10.7)		<.001
	Memory Lane				204.8 (44.8)		121.9 (6.1)		<.001

^a^AM, alcohol misuse.

^b^HD: heavy drinking.

^c^DD: drinking days.

^d^HDD: heavy drinking days.

^e^ACL: average craving level.

^f^HCL: highest craving level.

^g^PSA: processing speed and attention.

^h^WM: working memory.

### Feasibility and Acceptability of Brain+ Alco-Recover

Only 23.6% (n=17) of the patients completed the SUS at 6-month follow-up, but the overall experience with the Brain+ Alco-Recover app was well received by these patients in both groups, and no significant difference was found between the ratings of the 2 different versions of the app (*P*=.17). This was indicated by a good usability rating with a mean total score of 74.6 (SD 17.6) in the experimental group, while the sham group rated the usability of the training program as excellent with a mean total score of 81.5 (SD 11.9).

The criterion for determining the feasibility of the training program was reached in 5 out of the 6 games in the experimental version of the Brain+ Alco-Recover app, with a rate of patients completing the minimum requirements ranging from 51.4% to 82.9% (ie, corresponding to 18-29 participants) for the individual game type. The estimated average total time spent on the 1-month training program was 577 minutes for the experimental group and 919 minutes for the sham group (see Table S1 in Multimedia Appendix 1). The game that did not meet this criterion was Attention Island*,* where only 37.1% (n=13) of the patients completed the 5 required game trials. In the sham version of the app, the criterion was reached in 4 out of the 5 games. The game that did not meet the criterion was Bulky Codes, where 48% of the patients completed the 5 required game trials*.* There was no significant difference between the 2 groups regarding the number of completed game trials, except that significantly more patients in the sham group completed the minimum number of required game trials for Attention Island (n=13, 65.7%) compared to the experimental group (*P*=.04).

### Effects on Cognitive Outcomes

**Figure 2:**
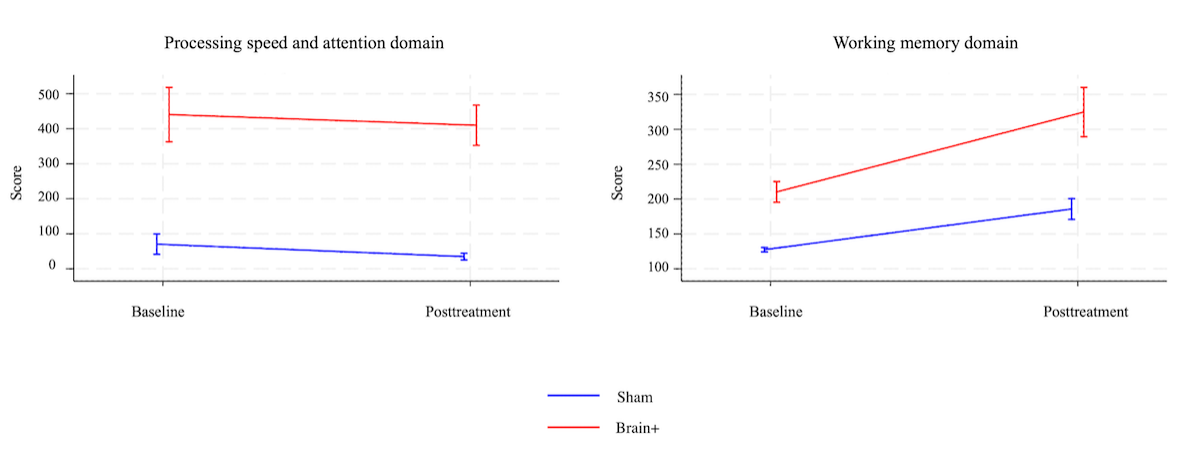
Change in mean domain scores from baseline to the posttreatment assessment for the experimental (ie, red line) and sham group (ie, blue line). A higher score indicates a better cognitive performance. The error bars depict the 95% CIs.

Comparisons between baseline and posttreatment assessments (see Figure 2) showed a minor improvement in PSA in both groups, with no indication of a group difference (*P*=.90). A significant difference was observed between the 2 groups in the working memory (WM) domain (*P*=.002). This difference favored the experimental group, which demonstrated a higher mean change score (WM: mean 114.6, 95% CI 82.1-147.1) compared to the sham group (WM: mean 58.3, 95% CI 43.9-72.7). This significant effect remained consistent in the PP-analyses after adjusting for patients who met the minimum training requirements (see Multimedia Appendix 1 for PP analyses).

### Effects on Alcohol Consumption and Craving

**Figure 3:**
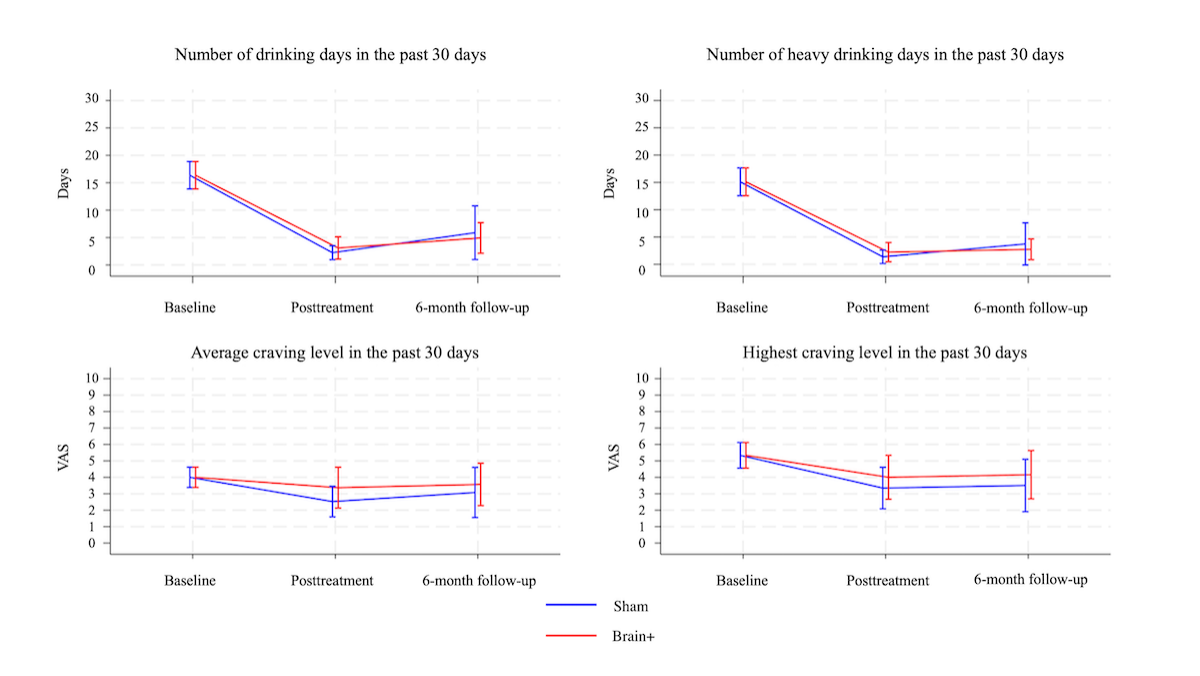
Mean change in number of drinking days, number of heavy drinking days, average craving, and highest craving level from baseline to posttreatment and 6-month follow-up. The error bars depict the 95% CIs. VAS: Visual Analogue Scale;.

No significant differences between baseline, posttreatment, and 6-month follow-up assessments were observed between 2 groups regarding DD, HDD, MCL, and HCL (see Figure 3 and Multimedia Appendix 1).

By inspecting the mean difference at 6-month follow-up, there was a pattern favoring the experimental group for both DD (mean –11.44, 95% CI –15.00 to –7.88) and HDD (mean –12.37, 95% CI –15.54 to –9.19) when compared to the sham group (DD: mean –10.48, 95% CI –16.10 to –4.86; HDD: mean –11.38, 95% CI –16.13 to –6.62).

## Discussion

### Principal Findings

The present feasibility and efficacy study provides the most recent evidence on the acceptability and usability of a semiguided, smartphone-based, serious gaming app administered as an add-on to conventional treatment among patients with AUD. The Brain+ Alco-Recover app was deemed feasible and accepted by the patients. Further, the experimental group exhibited significant improvements in WM compared to the sham group; however, these secondary effects should be interpreted with caution due to the in-game cognitive assessment. Our analyses did not reveal any significant differences between the 2 groups regarding alcohol consumption and craving, presumably due to inadequate power. Nonetheless, the observed reduction in alcohol consumption in the experimental group suggests that this type of cognitive training might be beneficial for clinical outcomes.

The adherence and acceptability of the cognitive training in this study are corroborated by previous findings in studies that examined training programs with gamified elements [15,16,20,21] and studies that delivered the training program using mHealth technology [15,16]. Nonetheless, it should be stressed that these results regarding the acceptability were based on our minimum criterion for the usage of the training program, which was lower compared to other studies that instructed the patients to complete 45-60-minute training sessions [16,20]. Despite our decision on using shorter, 20-minute sessions, still only one third of the patients adhered to the preinstructed training program. This could be indicative of fundamental issues with the game design of the training program and the means of administration. It has been proposed that a constant display of frustrating training elements or a display of negative scores may induce a state of failure and disbelief and thereby reduce the patients’ self-efficacy [24].

A reduction in self-efficacy could pose a potential threat to the effectiveness of the design of smartphone-based cognitive training programs, as one needs to balance the incentive to push the performance of the patients by constantly increasing the level of difficulty while ensuring that the patients receive an adequate amount of positive reinforcement. Although the differences in SUS scores should be interpreted with caution due to the low return rates, one could argue that the discrepancies between the two groups may be the result of one group encountering more challenging games. The lower SUS score in the experimental group could indicate that some of the games were perceived as too difficult, and thus patients could have experienced more frustration by being exposed to low scores and a high level of errors. In contrast, the level of difficulty in the sham version might have increased the completion rate, which in turn would decrease the number of self-perceived failures, resulting in more positive ratings on the SUS. This could highlight the importance of implementing the training into existing treatment programs, in which a health professional could encourage and coach the patient during the training program. Previous studies have shown that administering the cognitive training program in an environment in which the patient receives support to maintain his or her treatment goals can cultivate the effects of cognitive training [1,16,17]. Thus, balancing the optimal training conditions between a highly structured clinical setting and a more unguided approach could be imperative.

Albeit our findings of near-transfer improvements in WM were discovered using proprietary algorithms for assessment of cognition, they do corroborate findings from previous trials that demonstrated near-transfer effects using similar multifaceted cognitive training programs for AUD [18,23]. The lack of EF and episodic memory training in the sham version also means that our preliminary findings cannot be fully extended to a broader range of cognitive domains. However, other studies that examined cognitive training focusing specifically on WM were able to demonstrate far-transfer improvements in episodic memory [40,41]. It has also been suggested that cognitive functions do not work independently of each other, and tasks designed to train verbal memory are still highly dependent on the ability to direct one’s attentional resources to process and store information [2]. Thus, it could be hypothesized that even though the patients in the present study did not specifically train episodic memory, improvements in WM could manifest as improvements in other domains as well. However, relying solely on the in-game preassessment function for cognitive evaluation limits the ability to determine whether the observed near-transfer effects extend to distal domains not actively targeted by the current version of the Brain+ Alco-Recover app.

As expected, our findings showed that the cognitive training program did not result in statistically significant improvements in clinical outcomes. Notwithstanding, small and clinically relevant changes in alcohol consumption were observed. By inspecting the reduction in the mean number of DD and HDD, the experimental group did exhibit close to a 10% reduction in alcohol consumption.

Although a previous study by Kumar et al [18], with a comparable sample size (n=50) was able to detect improved abstinence rates, the cognitive training program was combined with yoga, breathing exercises, and psychoeducation. This resulted in a more guided intervention that could potentially have induced a synergistic effect of the different treatment elements. In contrast, other research groups have failed to demonstrate significant improvements in clinical outcomes [20,21,42,43], which corroborates the findings of the present study. Most of these studies included small sample sizes (n*≤*60), but one research group [20] did identify a trend for reduction in units per drinking day (*P*=.07). The training program used in the study by Khemiri et al [20], focused more on training tasks selectively targeting WM, and the patients also used the training program at home with minimal guidance. When aggregating these similarities, it could indicate that cognitive training programs, even when administered outside a treatment facility, may have clinically relevant effects among patients with AUD. However, the lack of evidence for clinical effects of the training program in the present study could also support the notion that cognitive training programs are too reductionistic to comprise the complexity needed for the induction of behavioral changes [2,12].

### Limitations

The present study has some limitations. First, the development of the Brain+ Alco-Recover app was in its early stages, and as a result, more complex features as well as adaptations of specific games were omitted. The lack of a notification system made it more difficult to remind the patients about their training sessions, and this could have impeded on the number of completed game trials and adherence to the training program. To mitigate the implications of this missing feature, we ensured that the clinicians at the outpatient facility reminded the patients to complete the training sessions.

Second, previously mentioned fundamental differences in the way that cognitive scores were calculated in the preassessment phase in the Brain+ Alco-Recover app hindered the patients using the sham version to attain cognitive scores comparable to those in the experimental group. As such, the observed baseline differences in cognitive performance between the two groups were expected and are unlikely to reflect allocation bias. Furthermore, since our primary outcome focused on within-group change from baseline to posttreatment, the method of cognitive score calculation is unlikely to have substantially affected the results in the sham group.

Third, the usage of the in-game assessment meant that we were unable to detect far-transfer effects. This would also preclude bold comparisons to the effects of other cognitive training programs that were assessed with standardized instruments, thus reducing the external validity of the present findings. Nonetheless, the precision and extendibility of the cognitive assessment and training algorithms implemented in the Brain+ app have been established in studies for acquired brain injury and Parkinson’s disease. Thus, it would be hypothesized that the findings in the present study could be indicative of benefits on cognitive performance.

Fourth, the present study did not examine the training effects on verbal learning and episodic memory due to technical restraints, making it impossible to cap the level of difficulty for the sham version. This should be addressed in future versions of the Brain+ Alco-Recover app, since learning and memory could be less susceptible to the spontaneous recovery occurring with continuing abstinence among patients with alcohol-related Wernicke-Korsakoff disorder [3]. However, as the included patients experienced less severe AUD and were not diagnosed with Wernicke-Korsakoff disorder, the additional benefits of implementing games targeting episodic memory could be questioned.

Last, as we in this study wanted to establish the usability and efficacy of the Brain+ app, it was not assumed that we could detect significant changes in the clinical outcomes between the experimental and sham groups. Therefore, the low sample size may have increased the risk of a Type 2. Nevertheless, in this study, potential improvement in clinical outcomes was examined to pave the way for a future, large-scaled trial, in which clinical effects could be ascertained.

### Conclusions

The use of the semiguided, Brain+ Alco-Recover serious gaming app was found to be a feasible and acceptable way of delivering cognitive training to patients with AUD in conjunction with conventional treatment. Although the potential near-transfer effects in WM should be interpreted with care, this early version, adapted to patients with AUD, could be beneficial for cognitive performance even when the training sessions were performed at home with minimal to no support from a health professional. The prospect of potential effects of the cognitive training program on alcohol consumption could have valuable clinical implications and highlights the importance of exploring the effects of the Brain+ app in large-scale trials. This would be an important step toward uncovering an effective way of ameliorating cognitive dysfunctions and improving clinical outcomes in the future treatment of AUD.

## Appendix

Multimedia Appendix 1 Supplementary material including screenshots of the cognitive games from Brain+ Alco-Recover and supplementary tables S1 and S2.

Multimedia Appendix 2 CONSORT-eHEALTH checklist (V 1.6.1).

## Abbreviations

ASI: Addiction Severity Index
AUD: alcohol use disorder
CONSORT: Consolidated Standards of Reporting Trials
CONSORT-EHEALTH: Consolidated Standards of Reporting Trials of Electronic and Mobile Health Applications and Online Telehealth
DD: drinking days
EF: executive functions
HCL: highest craving level
HD: heavy drinking
HDD: heavy drinking days
ICD-10: International Classification of Diseases the 10th edition
ITT: intention-to-treat
MCL: mean craving level
PP: per-protocol
PSA: processing speed and attention
SUD: substance use disorder
SUS: System Usability Scale
TAU: treatment-as-usual
VAS: Visual Analogue Scale
WM: working memory
