# Supplementary material for: A Personalized and Smartphone-Based Serious Gaming App Targeting Cognitive Impairments in Alcohol Use Disorder: Double-Blinded, Randomized Controlled Efficacy Trial Among Outpatients
Source: JMIR Ment Health. 2025 Oct 7;12:e67167. doi: 10.2196/67167 (PMC12541269; doi:10.2196/67167)
Supplement: Multimedia Appendix 1 [file mental_v12i1e67167_app1.pdf]

Multimedia appendix 1

Methods

In the eligibility criteria in the main paper, it was stated that we included participants between 18 and 70 years of age, this was a deviation from our eligibility criteria in our previously defined protocol [1]. Here the upper limit was set to 60 years, but due to unforeseen delays in the recruitment of participants, we increased the upper limit for the age to 70 years. However, in the final sample, only three participants older than 60 years were included (i.e., the oldest being 70 years).

Power calculation

The present study was a feasibility and efficacy trial, we decided not to conduct any formal power calculation. However, the goal specified in our pre-registered protocol was to include a minimum of 60 patients across the two groups to approach a normal distribution in accordance with the central limit theorem. Based on prior trials that examined cognitive training with small sample sizes [2-4], we expected an attrition rate of approximately 16% (n = 12 patients). Thus, 12 additional patients were recruited to ensure that we obtained the required sample size of 30 patients in each group. This resulted in a final sample size of 72 patients or 36 patients in each of the two groups.

Additional information on determining feasibility and acceptability

The minimum requirements (i.e., five completed game trials for each of the six games) determining our primary criterion for the feasibility outcome, were not conveyed to the patients, and they were only informed about the general instruction of the training program (i.e., 20 minutes a day, five days a week for one month). It was not possible to calculate the actual duration for the usage of the Brain+ Alco-Recover application throughout the training period, thus only the logged number of completed games was used as the outcome. Although the error in the game Pathfinder and the lack of a sham version of the game Remember Me meant that these games could not be used as assessments for executive functions and episodic memory, respectively, the number of completed game trials for both games was still logged for the entire training program. A bug in the application allowed the patients to skip certain games in a training block, thus we wanted to explore, how many patients completed at least 30 game trials regardless of the specific game type (i.e., meaning that the 30 completed games could consist of the same

one or two games, see table S1). Although this was not used for determining the feasibility of the training program, the number of patients in the experimental group adhering to the instructed training program was also noted (see table S2).

Specification of intervention, cognitive outcomes and process monitoring

The Brain+ application has been tested in multiple alternate versions by other research groups [5-7]. For the present trial, the Brain+ Recover application was adapted, a sham-version was created, and the two adapted versions were renamed Brain+ Alco-Recover. The developer team at Brain+ ensured that the training program in both the adapted versions consisted of the various types of games targeting multiple cognitive domains. In the experimental version of the Brain+ Alco-Recover, each time the patient successfully completed a game session, the results were visually presented, and the level of difficulty increased slightly to match the patient's current performance level. These incremental changes in difficulty included lower exposure time, additional items to recall, or more obstacles in the game, which would scale proportionally according to the patient's performance level. If the level of difficulty became too challenging and the patient could no longer complete the game session, the level of difficulty would decrease accordingly.

Six cognitive games were incorporated into the experimental version of the Brain+ Alco-Recover application. The game *Attention Island* (see figure S1 was categorized into attention (i.e., primarily targeting processing speed and visual attention), the game *Perception Speed* (see figure S2) was categorized into perception (i.e., primarily targeting visual perception and visual attention), the game *Pathfinder* (see figure S3) was categorized into logic (i.e., primarily targeting reasoning, problem solving, and planning skills), and finally, three games named *Bulky Codes* (see figure S4), *Memory Lane* (see figure S5), and *Remember Me* (see figure S6) were categorized into memory (i.e., primarily targeting visual working memory, visual learning, and episodic memory).

Since many of the games in the Brain+ application tapped into overlapping cognitive processes, the games were re-grouped across their pre-defined in-game categories. For the present study, the in-game category of memory was also split up so that working memory as well as learning and episodic memory could be assessed separately. Originally, the performance in four cognitive domains were pre-specified in our study protocol as executive functions (i.e., the

in-game Pathfinder), processing speed and attention (i.e., the in-games Perception Speed and Attention Island), working memory (i.e., the in-games Memory Lane and Bulky Codes), and finally learning and episodic memory (i.e., the in-game Remember Me). Bugs in the Brain+ application as well as difficulties with the implementation of a sham-version of the games for executive functions and episodic memory meant that the performance in these domains could not be assessed for the sham group. Thus, the executive and episodic memory domains were omitted, and the primary outcomes reported in this study was on two domains: processing speed/attention and working memory.

Adhering to the instructions of 20 minutes a day, five days a week, for one month, would result in the completion of 20 training sessions (i.e., 40 training blocks) and this was equal to 150 games, which meant that each of the six different games appeared 25 times. These were the minimum requirements, and the patients were free to train more frequently if they wanted. During the study, a bug was discovered in the application that caused a game to skip (i.e., not marked as completed) if the patient closed the application in the middle of playing that game, and the next game in the training block would then appear. This meant that the patients could play certain games more frequently than others resulting in an uneven distribution of completed games across the training blocks. To monitor the effects of this bug, the actual number of completed games was logged throughout the entire training period.

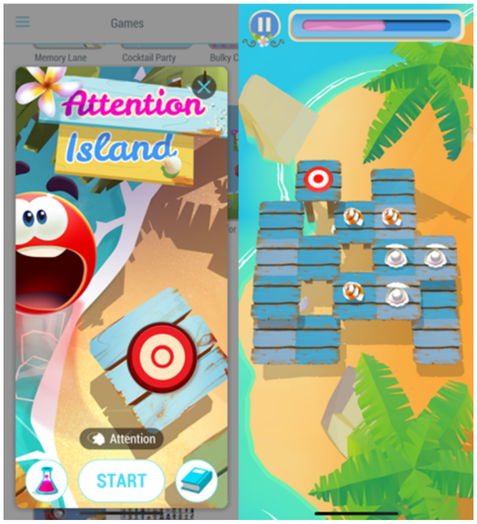

Figure S1. A sample of the game *Attention Island* that trains visual attention and short-term memory. This game was categorized into the domain for processing speed and attention. Here the user is presented with white shells in various places for a brief amount of time. The task is to guide the red character to the designated area and then select the placement of the white shells.

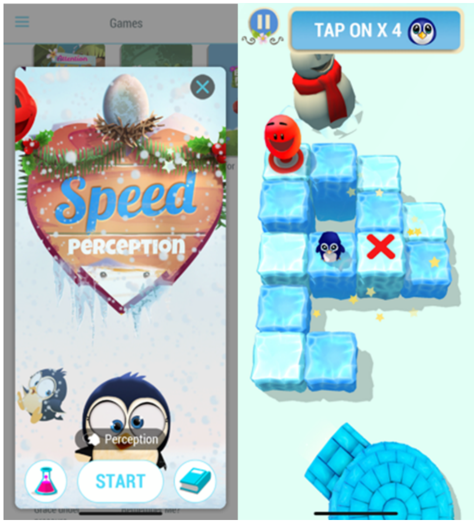

Figure S2. A sample of the game *Speed Perception* that trains visual attention and short-term memory. This game was categorized into the domain for processing speed and attention. Here the task is to remember the placement of the penguins which are only presented for a brief amount of time. The user must guide the red character to the targeted area and select the correct placement of the penguins.

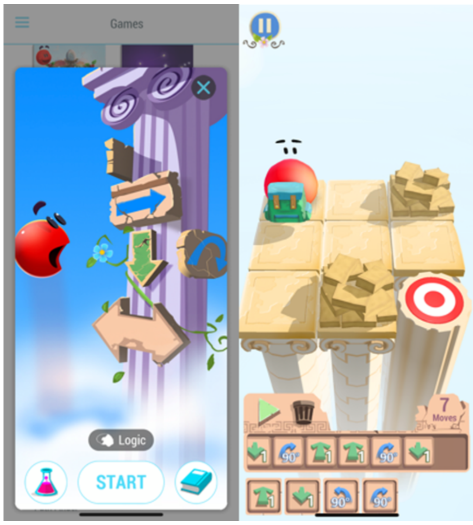

Figure S3. A sample of the game *Path Finder* that trains logic and problem-solving abilities. This game was categorized into executive functions. Here the task is to plan the right path for the red character, so it reaches the red target. This is done by using a combination of movement operators. The character will only move when the play button is pressed.

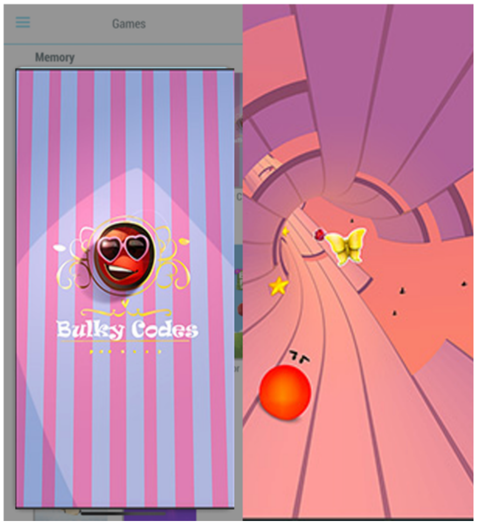

Figure S4. A sample of the game *Bulky Codes* that trains visual working memory (WM). This game was categorized into the domain for WM. The task consists of remembering and collecting the correct symbols (e.g., banana, flower, watermelon) in the correct order.

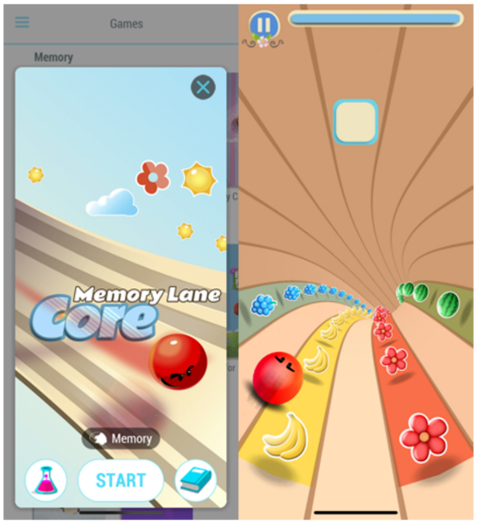

Figure S5. A sample of the game *Memory Lane* that trains visual working memory (WM). This game was categorized into the domain for WM. The task consists of remembering and collecting the correct symbols (e.g., banana, flower, watermelon) in the correct order.

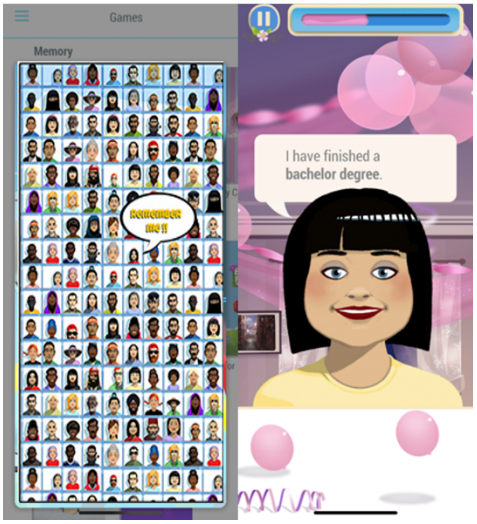

Figure S6. A sample of the game *Remember Me?* that trains visual learning. Thus, game was categorized into learning and episodic memory. Here the task is to remember the names of different characters and remember specific details that are presented throughout the game. This game was not possible to implement into the sham version, thus only the experimental group was presented with this game.

Results

This section includes additional descriptive statistics on game usage, ITT- and PP-analyses on cognitive outcomes as well as extended results for our exploratory analyses on alcohol consumption and craving.

Feasibility and acceptability outcomes

The number of patients completing the minimum number of required games for each game type reached the 50% criterion for feasibility in all games except for Attention Island (P = .28; see table S1). Disregarding the individual game types, the number of patients completing 30 game trials or more was at 74.3% in the experimental group (n = 26) and 66.7% in the sham group (n = 24). The average number of game trials completed by the patients fulfilling this criterion was 217.4 trials in the experimental group (i.e., estimated to a total of 577 minutes of training time) and 345.6 trials in the sham group (i.e., estimated to a total of 919 minutes of training time). There was no significant difference between the groups (P = .61).

When focusing on the instructed training program the number of patients completing the required 25 games or more was low across all games except for Pathfinder, which was completed by 54.3% of the patients in the experimental group (n = 19) and 44.4% of the patients in the sham group (n = 16). This was also one of the games that, where a significant difference was observed between the two groups (P = .02). Furthermore, a significant difference for the groups was also present for Attention Island (P < .001).

Intention-to-treat-analyses for cognitive outcomes for individual games

To explore possible effects on the cognitive performance for the individual games, we conducted analyses for each game type. Here we only found significant differences for *Bulky Codes* (P < .001) and *Memory Lane* (P < .001), which were driven by improvements in the experimental group (i.e., *Bulky Codes: M* = 111.0, 95% CI [75.4; 146.6] and *Memory Lane: M* = 141.2, 95% CI [108.3; 174.2]) compared to the improvement in the sham group (i.e., *Bulky Codes: M* = 59.3, 95% CI [52.8; 65.7] and *Memory Lane: M* = 65.4, 95% CI [48.8; 82.0]).

Per-protocol analyses for cognitive outcomes

We conducted per-protocol analyses for our cognitive outcomes, adjusting for patients who met the requirement of completing at least five game trials. The significant effects observed in the intention-to-treat analyses remained non-significant for processing speed and attention (P < .739) but continued to be significant for working memory (P < .001). This significant difference was primarily driven by higher mean change scores in the experimental group (working memory: M = 143.6, 95% CI [107.1; 180.1]) compared to the sham group (working memory: M = 68.6, 95% CI [62.1; 75.2]). Further examination of improvements in cognitive performance on individual games revealed significant mean changes favoring the experimental group in the following tasks: Attention Island (M = 29.1, 95% CI [22.6; 35.5], P < .001) compared to the sham group (M = -2.6, 95% CI [-5.0; -0.1]), Bulky Codes (experimental group: M = 171.2, 95% CI [136.8; 205.6], P < .001, compared to the sham group: M = 80.9, 95% CI [77.0; 84.9]), and Memory Lane (experimental group: M = 158.4, 95% CI [116.5; 200.3], P < .001, compared to the sham group: M = 29.1, 95% CI [22.6; 35.5]).

Table S1. Number of patients minimum requirements for usage of Brain+ Alco-Recover

	Experimental	Sham	
	n = 35	*n* = 36	*P* value
**Completed 5 ≤ game trials for individual game type, *n* (%)**			
**Bulky Codes**	18 (51.4)	17 (47.2)	.81
**Memory Lane**	24 (68.6)	20 (55.6)	.46
**Attention Island**	13 (37.1)	23 (63.9)	.03
**Speed Perception**	28 (80.0)	24 (66.7)	.28
**Pathfinder**	29 (82.9)	24 (66.7)	.26
**Remember Me**	28 (80.0)	*N/A*	*N/A*
**Completed 30 ≤ game trials irrespective of game type, *n* (%)**	26 (74.3)	24 (66.7)	.61

Note. The number of patients in each group meeting the minimum requirements for the training program, which was set to a minimum of five game trials for each game type. As some game types could be accidentally skipped, the number of patients in each group completing 30 game trials irrespective of specific game type was specified as well. All analyses were conducted using Fisher's Exact test and by principles of intention-to-treat. There were no completers for the game *Remember Me* in the sham group because this game could only be implemented in the experimental version. N/A, not applicable; SD, standard deviation.

Table S2. Number of patients adhering to the instructed training program

	Experimental	Sham	
	n = 35	*n* = 36	*P* value
**Bulky Codes, *n* (%)**	10 (28.6)	10 (28.6)	1.0
**Memory Lane, *n* (%)**	6 (17.1)	9 (25.0)	.56
**Attention Island, *n* (%)**	5 (14.3)	20 (55.6)	< .001
**Speed Perception, *n* (%)**	13 (37.1)	16 (44.4)	.63
**Pathfinder, *n* (%)**	19 (54.3)	16 (44.4)	.02
**Remember Me, *n* (%)**	6 (17.1)	*N/A*	*N/A*
**Completed 150 ≤ game trials irrespective of game type**	11 (31.4)	12 (33.3)	1.00

Note. The pre-instructed training program consisted of completing a minimum of 25 game trials for a single game type. All analyses were conducted using Fisher's Exact test and by principles of intention-to-treat. There were no completers for the game *Remember Me* in the sham group because this game could only be implemented in the experimental version. N/A, not applicable; SD, standard deviation.

Exploratory analyses for effects on alcohol consumption and craving

No significant effects were found between the groups from baseline to post-treatment in alcohol consumption (drinking days [DD]: *M* = 0.87, 95% CI [-1.54; 3.27], *P* = 0.48; heavy drinking days [HDD]: *M* = 0.85, 95% CI [-1.30; 3.01], *P* = .44) when comparing the experimental (*M* = -13.27, 95% CI [-16.53; -10.00]) and the sham group (*M* = -14.13, 95% CI [-16.67; -11.59]). No significant effect was evident for the mean craving level (MCL: 0.85, 95% CI [-0.69; 2.39], *P* = .28) when comparing the experimental group (*M* = -12.88, 95% CI [-15.98; -9.78]) with sham (*M* = -13.74, 95% CI [-16.32; -11.15]). The comparison between the baseline and the 6-months follow-up assessment revealed a similar pattern, with no significant differences between the two groups regarding DD (*M* = -0.96, 95% CI [-6.62; 4.70], *P* = .74), HDD (*M* = -0.99, 95% CI [-5.32; 3.33], *P* = .65), MCL (*M* = 0.49, 95% CI [-1.47; 2.45], *P* = .62), and highest craving level (*M* = 0.66, 95% CI [-1.48; 2.79], *P* = .55).

References

1. Mistarz, N., et al., *Brain+ AlcoRecover: A Randomized Controlled Pilot-Study and Feasibility Study of Multiple-Domain Cognitive Training Using a Serious Gaming App for Treating Alcohol Use Disorders.* Frontiers in Psychiatry, 2021. **12**: p. 727001.
2. Bell, M.D., N.A. Vissicchio, and A.J. Weinstein, *Cognitive Training and Work Therapy for the Treatment of Verbal Learning and Memory Deficits in Veterans With Alcohol Use Disorders.* Journal of Dual Diagnosis, 2016. **12**(1): p. 83-89.
3. Khemiri, L., et al., *Working Memory Training in Alcohol Use Disorder: A Randomized Controlled Trial.* Alcoholism: Clinical and Experimental Research, 2019. **43**(1): p. 135-146.
4. Rupp, C.I., et al., *Cognitive Remediation Therapy During Treatment for Alcohol Dependence.* Journal of Studies on Alcohol and Drugs, 2012. **73**(4): p. 625-634.
5. Svaerke, K., et al., *Effects of Computer-Based Cognitive Rehabilitation on Attention, Executive Functions, and Quality of Life in Patients with Parkinson's Disease: A Randomized, Controlled, Single-Blinded Pilot Study.* Dementia and Geriatric Cognitive Disorders, 2021. **50**(6): p. 519-528.
6. Svaerke, K., et al., *Effects of computer-based cognitive rehabilitation on working memory in patients with acquired brain injury in the chronic phase, a pilot-study.* Brain Injury, 2022. **36**(4): p. 503-513.
7. Landowska, A., et al., *Adaptative computerized cognitive training decreases mental workload during working memory precision task - A preliminary fNIRS study.* International Journal of Human-Computer Studies, 2024. **184**: p. 103206.
