# Supplementary material for: A Personalized and Smartphone-Based Serious Gaming App Targeting Cognitive Impairments in Alcohol Use Disorder: Double-Blinded, Randomized Controlled Efficacy Trial Among Outpatients
Source: JMIR Ment Health. 2025 Oct 7;12:e67167. doi: 10.2196/67167 (PMC12541269; doi:10.2196/67167)
Supplement: Multimedia Appendix 2 [file mental_v12i1e67167_app2.pdf]

CONSORT-EHEALTH (V 1.6.1) - Submission/Publication Form

The CONSORT-EHEALTH checklist is intended for authors of randomized trials evaluating web-based and Internet-based applications/interventions, including mobile interventions, electronic games (incl multiplayer games), social media, certain telehealth applications, and other interactive and/or networked electronic applications. Some of the items (e.g. all subitems under item 5 - description of the intervention) may also be applicable for other study designs.

The goal of the CONSORT EHEALTH checklist and guideline is to be
a) a guide for reporting for authors of RCTs,
b) to form a basis for appraisal of an ehealth trial (in terms of validity)

CONSORT-EHEALTH items/subitems are MANDATORY reporting items for studies published in the Journal of Medical Internet Research and other journals / scientific societies endorsing the checklist.

Items numbered 1., 2., 3., 4a., 4b etc are original CONSORT or CONSORT-NPT (non-pharmacologic treatment) items.
Items with Roman numerals (i., ii, iii, iv etc.) are CONSORT-EHEALTH extensions/clarifications.

As the CONSORT-EHEALTH checklist is still considered in a formative stage, we would ask that you also RATE ON A SCALE OF 1-5 how important/useful you feel each item is FOR THE PURPOSE OF THE CHECKLIST and reporting guideline (optional).

Mandatory reporting items are marked with a red *.
In the textboxes, either copy & paste the relevant sections from your manuscript into this form - please include any quotes from your manuscript in QUOTATION MARKS,
or answer directly by providing additional information not in the manuscript, or elaborating on why the item was not relevant for this study.

YOUR ANSWERS WILL BE PUBLISHED AS A SUPPLEMENTARY FILE TO YOUR PUBLICATION IN JMIR AND ARE CONSIDERED PART OF YOUR PUBLICATION (IF ACCEPTED).
Please fill in these questions diligently. Information will not be copyedited, so please use proper spelling and grammar, use correct capitalization, and avoid abbreviations.

DO NOT FORGET TO SAVE AS PDF _AND_ CLICK THE SUBMIT BUTTON SO YOUR ANSWERS ARE IN OUR DATABASE !!!

Citation Suggestion (if you append the pdf as Appendix we suggest to cite this paper in the caption):
Eysenbach G, CONSORT-EHEALTH Group
CONSORT-EHEALTH: Improving and Standardizing Evaluation Reports of Web-based and Mobile Health Interventions
J Med Internet Res 2011;13(4):e126
URL: http://www.jmir.org/2011/4/e126/
doi: 10.2196/jmir.1923
PMID: 22209829

nicolaj.mstar@gmail.com Switch accounts

Not shared

Draft saved

* Indicates required question

Your name *

First Last

Nicolaj Mistarz

Primary Affiliation (short), City, Country *

University of Toronto, Toronto, Canada

University of Southern Denmark

Your e-mail address *

abc@gmail.com

nimistarz@health.sdu.dk

Title of your manuscript *

Provide the (draft) title of your manuscript.

A personalized and smartphone-based, serious gaming applica-tion targeting cognitive impairments in alcohol use disorder: A piloting, double-blinded, randomized controlled trial

Name of your App/Software/Intervention *

If there is a short and a long/alternate name, write the short name first and add the long name in brackets.

Brain+ Alco-Recover

Evaluated Version (if any)

e.g. "V1", "Release 2017-03-01", "Version 2.0.27913"

Beta-version [specifically developed for the pre

Language(s) *

What language is the intervention/app in? If multiple languages are available, separate by comma (e.g. "English, French")

Danish, English

URL of your Intervention Website or App

e.g. a direct link to the mobile app on app in appstore (itunes, Google Play), or URL of the website. If the intervention is a DVD or hardware, you can also link to an Amazon page.

https://www.brain-plus.com

URL of an image/screenshot (optional)

Your answer

Accessibility *

Can an enduser access the intervention presently?

- ○ access is free and open
- ○ access only for special usergroups, not open
- ○ access is open to everyone, but requires payment/subscription/in-app purchases
- ○ app/intervention no longer accessible
- ◉ Other: Other versions of the Brain+ platform may still be available upon valid

Primary Medical Indication/Disease/Condition *

e.g. "Stress", "Diabetes", or define the target group in brackets after the condition, e.g. "Autism (Parents of children with)", "Alzheimers (Informal Caregivers of)"

Alcohol Use Disorder

Primary Outcomes measured in trial *

comma-separated list of primary outcomes reported in the trial

Cognitive performance

Secondary/other outcomes

Are there any other outcomes the intervention is expected to affect?

Alcohol consumption and craving

Recommended "Dose" *

What do the instructions for users say on how often the app should be used?

- (x) Approximately Daily
- ( ) Approximately Weekly
- ( ) Approximately Monthly
- ( ) Approximately Yearly
- ( ) "as needed"
- ( ) Other:

Approx. Percentage of Users (starters) still using the app as recommended after 3 months *

- (x) unknown / not evaluated
- ( ) 0-10%
- ( ) 11-20%
- ( ) 21-30%
- ( ) 31-40%
- ( ) 41-50%
- ( ) 51-60%
- ( ) 61-70%
- ( ) 71%-80%
- ( ) 81-90%
- ( ) 91-100%
- ( ) Other:

Overall, was the app/intervention effective? *

- ○ yes: all primary outcomes were significantly better in intervention group vs control
- ◉ partly: SOME primary outcomes were significantly better in intervention group vs control
- ○ no statistically significant difference between control and intervention
- ○ potentially harmful: control was significantly better than intervention in one or more outcomes
- ○ inconclusive: more research is needed
- ○ Other:

Article Preparation Status/Stage *

At which stage in your article preparation are you currently (at the time you fill in this form)

- ○ not submitted yet - in early draft status
- ○ not submitted yet - in late draft status, just before submission
- ◉ submitted to a journal but not reviewed yet
- ○ submitted to a journal and after receiving initial reviewer comments
- ○ submitted to a journal and accepted, but not published yet
- ○ published
- ○ Other:

Journal *

If you already know where you will submit this paper (or if it is already submitted), please provide the journal name (if it is not JMIR, provide the journal name under "other")

- ◯ not submitted yet / unclear where I will submit this
- ◯ Journal of Medical Internet Research (JMIR)
- ◯ JMIR mHealth and UHealth
- ◯ JMIR Serious Games
- ◉ JMIR Mental Health
- ◯ JMIR Public Health
- ◯ JMIR Formative Research
- ◯ Other JMIR sister journal
- ◯ Other:

Is this a full powered effectiveness trial or a pilot/feasibility trial? *

- ◉ Pilot/feasibility
- ◯ Fully powered

Manuscript tracking number *

If this is a JMIR submission, please provide the manuscript tracking number under "other" (The ms tracking number can be found in the submission acknowledgement email, or when you login as author in JMIR. If the paper is already published in JMIR, then the ms tracking number is the four-digit number at the end of the DOI, to be found at the bottom of each published article in JMIR)

- (x) no ms number (yet) / not (yet) submitted to / published in JMIR
- ( ) Other:

TITLE AND ABSTRACT

1a) TITLE: Identification as a randomized trial in the title

1a) Does your paper address CONSORT item 1a? *

I.e does the title contain the phrase "Randomized Controlled Trial"? (if not, explain the reason under "other")

- (x) yes
- ( ) Other:

1a-i) Identify the mode of delivery in the title

Identify the mode of delivery. Preferably use “web-based” and/or “mobile” and/or “electronic game” in the title. Avoid ambiguous terms like “online”, “virtual”, “interactive”. Use “Internet-based” only if Intervention includes non-web-based Internet components (e.g. email), use “computer-based” or “electronic” only if offline products are used. Use “virtual” only in the context of “virtual reality” (3-D worlds). Use “online” only in the context of “online support groups”. Complement or substitute product names with broader terms for the class of products (such as “mobile” or “smart phone” instead of “iphone”), especially if the application runs on different platforms.

	1	2	3	4	5	
subitem not at all important	○	○	○	○	◉	essential

Clear selection

Does your paper address subitem 1a-i? *

Copy and paste relevant sections from manuscript title (include quotes in quotation marks "like this" to indicate direct quotes from your manuscript), or elaborate on this item by providing additional information not in the ms, or briefly explain why the item is not applicable/relevant for your study

"A personalized and smartphone-based, serious gaming application [...]"

1a-ii) Non-web-based components or important co-interventions in title

Mention non-web-based components or important co-interventions in title, if any (e.g., “with telephone support”).

	1	2	3	4	5	
subitem not at all important	◉	○	○	○	○	essential

Clear selection

Does your paper address subitem 1a-ii?

Copy and paste relevant sections from manuscript title (include quotes in quotation marks "like this" to indicate direct quotes from your manuscript), or elaborate on this item by providing additional information not in the ms, or briefly explain why the item is not applicable/relevant for your study

Our intervention is only delivered through a smartphone application

1a-iii) Primary condition or target group in the title

Mention primary condition or target group in the title, if any (e.g., "for children with Type I Diabetes") Example: A Web-based and Mobile Intervention with Telephone Support for Children with Type I Diabetes: Randomized Controlled Trial

	1	2	3	4	5	
subitem not at all important	○	○	○	○	●	essential

Clear selection

Does your paper address subitem 1a-iii? *

Copy and paste relevant sections from manuscript title (include quotes in quotation marks "like this" to indicate direct quotes from your manuscript), or elaborate on this item by providing additional information not in the ms, or briefly explain why the item is not applicable/relevant for your study

"[...] cognitive impairments in alcohol use disorder"

1b) ABSTRACT: Structured summary of trial design, methods, results, and conclusions

NPT extension: Description of experimental treatment, comparator, care providers, centers, and blinding status.

1b-i) Key features/functionalities/components of the intervention and comparator in the METHODS section of the ABSTRACT

Mention key features/functionalities/components of the intervention and comparator in the abstract. If possible, also mention theories and principles used for designing the site. Keep in mind the needs of systematic reviewers and indexers by including important synonyms. (Note: Only report in the abstract what the main paper is reporting. If this information is missing from the main body of text, consider adding it)

	1	2	3	4	5	
subitem not at all important	○	○	○	○	◉	essential

Clear selection

Does your paper address subitem 1b-i? *

Copy and paste relevant sections from the manuscript abstract (include quotes in quotation marks "like this" to indicate direct quotes from your manuscript), or elaborate on this item by providing additional information not in the ms, or briefly explain why the item is not applicable/relevant for your study

"[...] Group A) experimental + TAU (n = 36), or Group B) sham + TAU (n = 36), and they had to complete a 1-month training program in addition to primary treatment."

1b-ii) Level of human involvement in the METHODS section of the ABSTRACT

Clarify the level of human involvement in the abstract, e.g., use phrases like "fully automated" vs. "therapist/nurse/care provider/physician-assisted" (mention number and expertise of providers involved, if any). (Note: Only report in the abstract what the main paper is reporting. If this information is missing from the main body of text, consider adding it)

	1	2	3	4	5	
subitem not at all important	○	○	◉	○	○	essential

Clear selection

Does your paper address subitem 1b-ii?

Copy and paste relevant sections from the manuscript abstract (include quotes in quotation marks "like this" to indicate direct quotes from your manuscript), or elaborate on this item by providing additional information not in the ms, or briefly explain why the item is not applicable/relevant for your study

The smartphone application is a part of primary treatment, where the patients are not assisted or guided from their health care practitioners

1b-iii) Open vs. closed, web-based (self-assessment) vs. face-to-face assessments in the METHODS section of the ABSTRACT

Mention how participants were recruited (online vs. offline), e.g., from an open access website or from a clinic or a closed online user group (closed usergroup trial), and clarify if this was a purely web-based trial, or there were face-to-face components (as part of the intervention or for assessment). Clearly say if outcomes were self-assessed through questionnaires (as common in web-based trials). Note: In traditional offline trials, an open trial (open-label trial) is a type of clinical trial in which both the researchers and participants know which treatment is being administered. To avoid confusion, use “blinded” or “unblinded” to indicated the level of blinding instead of “open”, as “open” in web-based trials usually refers to “open access” (i.e. participants can self-enrol). (Note: Only report in the abstract what the main paper is reporting. If this information is missing from the main body of text, consider adding it)

	1	2	3	4	5	
subitem not at all important	○	○	○	○	◉	essential

Clear selection

Does your paper address subitem 1b-iii?

Copy and paste relevant sections from the manuscript abstract (include quotes in quotation marks "like this" to indicate direct quotes from your manuscript), or elaborate on this item by providing additional information not in the ms, or briefly explain why the item is not applicable/relevant for your study

"A total of 72 treatment-seeking patients from an outpatient facility were blinded and randomized into either [...]"

1b-iv) RESULTS section in abstract must contain use data

Report number of participants enrolled/assessed in each group, the use/uptake of the intervention (e.g., attrition/adherence metrics, use over time, number of logins etc.), in addition to primary/secondary outcomes. (Note: Only report in the abstract what the main paper is reporting. If this information is missing from the main body of text, consider adding it)

	1	2	3	4	5	
subitem not at all important	○	○	○	○	◉	essential

Clear selection

Does your paper address subitem 1b-iv?

Copy and paste relevant sections from the manuscript abstract (include quotes in quotation marks "like this" to indicate direct quotes from your manuscript), or elaborate on this item by providing additional information not in the ms, or briefly explain why the item is not applicable/relevant for your study

"[...] Group A) experimental + TAU (n = 36), or Group B) sham + TAU (n = 36), and they had to complete a 1-month training program in addition to primary treatment."

1b-v) CONCLUSIONS/DISCUSSION in abstract for negative trials

Conclusions/Discussions in abstract for negative trials: Discuss the primary outcome - if the trial is negative (primary outcome not changed), and the intervention was not used, discuss whether negative results are attributable to lack of uptake and discuss reasons. (Note: Only report in the abstract what the main paper is reporting. If this information is missing from the main body of text, consider adding it)

	1	2	3	4	5	
subitem not at all important	○	○	◉	○	○	essential

Clear selection

Does your paper address subitem 1b-v?

Copy and paste relevant sections from the manuscript abstract (include quotes in quotation marks "like this" to indicate direct quotes from your manuscript), or elaborate on this item by providing additional information not in the ms, or briefly explain why the item is not applicable/relevant for your study

"The smartphone-based, serious gaming application showed promising effects for improving cognitive functions when delivered as add-on to TAU, but a larger scaled trial should be conducted in the future to assess its potential clinical effectiveness."

INTRODUCTION

2a) In INTRODUCTION: Scientific background and explanation of rationale

2a-i) Problem and the type of system/solution

Describe the problem and the type of system/solution that is object of the study: intended as stand-alone intervention vs. incorporated in broader health care program? Intended for a particular patient population? Goals of the intervention, e.g., being more cost-effective to other interventions, replace or complement other solutions? (Note: Details about the intervention are provided in “Methods” under 5)

	1	2	3	4	5	
subitem not at all important	○	○	○	○	●	essential

Clear selection

Does your paper address subitem 2a-i? *

Copy and paste relevant sections from the manuscript (include quotes in quotation marks "like this" to indicate direct quotes from your manuscript), or elaborate on this item by providing additional information not in the ms, or briefly explain why the item is not applicable/relevant for your study

"The main aim of this pilot and feasibility study was to examine whether an unguided, smartphone-based, serious gaming application administered as add-on to conventional treatment [...]"

2a-ii) Scientific background, rationale: What is known about the (type of) system

Scientific background, rationale: What is known about the (type of) system that is the object of the study (be sure to discuss the use of similar systems for other conditions/diagnoses, if appropiate), motivation for the study, i.e. what are the reasons for and what is the context for this specific study, from which stakeholder viewpoint is the study performed, potential impact of findings [2]. Briefly justify the choice of the comparator.

	1	2	3	4	5	
subitem not at all important	○	○	○	○	◉	essential

Clear selection

Does your paper address subitem 2a-ii? *

Copy and paste relevant sections from the manuscript (include quotes in quotation marks "like this" to indicate direct quotes from your manuscript), or elaborate on this item by providing additional information not in the ms, or briefly explain why the item is not applicable/relevant for your study

"A few studies focusing on domain-focused cognitive training 27-29 have examined the effects on alcohol consumption, but no significant reductions were found when compared to the control groups. Contrasting the findings for domain-focused programs, three studies utilized a training program targeting multiple domains 22,24,25, and two of these studies found that the intervention improved the rate of abstinence among the patients 22,25."

2b) In INTRODUCTION: Specific objectives or hypotheses

Does your paper address CONSORT subitem 2b? *

Copy and paste relevant sections from the manuscript (include quotes in quotation marks "like this" to indicate direct quotes from your manuscript), or elaborate on this item by providing additional information not in the ms, or briefly explain why the item is not applicable/relevant for your study

"The main aim of this pilot and feasibility study was to examine whether an unguided, smartphone-based, serious gaming application administered as add-on to conventional treatment can improve both cognitive and alcohol-related outcomes among patients with AUD. The secondary aim was to assess the acceptability and feasibility of the training program."

METHODS

3a) Description of trial design (such as parallel, factorial) including allocation ratio

Does your paper address CONSORT subitem 3a? *

Copy and paste relevant sections from the manuscript (include quotes in quotation marks "like this" to indicate direct quotes from your manuscript), or elaborate on this item by providing additional information not in the ms, or briefly explain why the item is not applicable/relevant for your study

"This pilot trial was conducted as a double-blinded, randomized controlled trial (RCT) with two parallel groups. An urn randomization technique was used without any stratification 42, and the allocation of patients to the groups was performed by an independent statistician."

3b) Important changes to methods after trial commencement (such as eligibility criteria), with reasons

Does your paper address CONSORT subitem 3b? *

Copy and paste relevant sections from the manuscript (include quotes in quotation marks "like this" to indicate direct quotes from your manuscript), or elaborate on this item by providing additional information not in the ms, or briefly explain why the item is not applicable/relevant for your study

"No amendments were made to the original protocol, but minor changes to the total sample and age of the participants were incorporated into the study."

"Bugs in the Brain+ application as well as difficulties with the implementation of a sham-version of the games for executive functions and episodic memory meant that the performance in these domains could not be assessed for the sham group. Thus, the executive and episodic memory domains were omitted, and the primary outcomes reported in this study was on two domains: processing speed/attention and working memory."

3b-i) Bug fixes, Downtimes, Content Changes

Bug fixes, Downtimes, Content Changes: ehealth systems are often dynamic systems. A description of changes to methods therefore also includes important changes made on the intervention or comparator during the trial (e.g., major bug fixes or changes in the functionality or content) (5-iii) and other “unexpected events” that may have influenced study design such as staff changes, system failures/downtimes, etc. [2].

	1	2	3	4	5	
subitem not at all important	○	○	○	○	◉	essential

Clear selection

Does your paper address subitem 3b-i?

Copy and paste relevant sections from the manuscript (include quotes in quotation marks "like this" to indicate direct quotes from your manuscript), or elaborate on this item by providing additional information not in the ms, or briefly explain why the item is not applicable/relevant for your study

"During the study, a bug was discovered in the application that caused a game to skip (i.e., not marked as completed) if the patient closed the application in the middle of playing that game, and the next game in the training block would then appear. This meant that the patients could play certain games more frequently than others resulting in an uneven distribution of completed games across the training blocks. To monitor the effects of this bug, the actual number of completed games was logged throughout the entire training period."

4a) Eligibility criteria for participants

Does your paper address CONSORT subitem 4a? *

Copy and paste relevant sections from the manuscript (include quotes in quotation marks "like this" to indicate direct quotes from your manuscript), or elaborate on this item by providing additional information not in the ms, or briefly explain why the item is not applicable/relevant for your study

"Patients were eligible to participate in the study if they: 1) had a confirmed diag-nosis of AUD (i.e., harmful use/abuse or dependence); 2) had access to a smartphone or tablet that met the requirements for using the gamified cognitive training application; 3) were between 18 and 70 years of age; 4) spoke Danish; 5) provided informed consent to participate; and 6) had completed detoxification prior to study inclusion if deemed necessary. Patients were excluded if they had a diagnosis of SUD other than AUD, a severe mental disorder with suicidal, manic or psychotic symptoms, a neurological disorder (e.g., acquired brain injury, dementia, Parkinson's disease) including motoric impairments that can challenge or impede performance in the cognitive training program, or a terminal illness."

4a-i) Computer / Internet literacy

Computer / Internet literacy is often an implicit “de facto” eligibility criterion - this should be explicitly clarified.

	1	2	3	4	5	
subitem not at all important	○	○	○	○	●	essential

Clear selection

Does your paper address subitem 4a-i?

Copy and paste relevant sections from the manuscript (include quotes in quotation marks "like this" to indicate direct quotes from your manuscript), or elaborate on this item by providing additional information not in the ms, or briefly explain why the item is not applicable/relevant for your study

2) had access to a smartphone or tablet that met the requirements for using the gamified cognitive training application;

4a-ii) Open vs. closed, web-based vs. face-to-face assessments:

Open vs. closed, web-based vs. face-to-face assessments: Mention how participants were recruited (online vs. offline), e.g., from an open access website or from a clinic, and clarify if this was a purely web-based trial, or there were face-to-face components (as part of the intervention or for assessment), i.e., to what degree got the study team to know the participant. In online-only trials, clarify if participants were quasi-anonymous and whether having multiple identities was possible or whether technical or logistical measures (e.g., cookies, email confirmation, phone calls) were used to detect/prevent these.

	1	2	3	4	5	
subitem not at all important	○	○	○	○	◉	essential

Clear selection

Does your paper address subitem 4a-ii? *

Copy and paste relevant sections from the manuscript (include quotes in quotation marks "like this" to indicate direct quotes from your manuscript), or elaborate on this item by providing additional information not in the ms, or briefly explain why the item is not applicable/relevant for your study

"During the primary treatment, a healthcare professional conducted a clinical as-sessment of the patients. This included an assessment of AUD and other substance use disorders (SUD) as well as severe mental (e.g., suicidal, manic, or psychotic symptoms), neurological (e.g., cognitive or motor symptoms that can challenge or impede performance in the cognitive training program), and somatic disorders ac-cording to the 10th version of the International Classification of Diseases (ICD-10) 45."

"After the initial clinical baseline interview and prior to initiating the primary treat-ment at the outpatient clinic (i.e., just after completing the detoxification program if deemed necessary), eligible patients were informed about the study by their health care providers. Patients who expressed interest in participating were re-ferred to a research assistant who provided verbal and written information about the study following a standardized procedure. The research assistant scheduled a meeting with those patients who agreed to participate, which took place at the ear-liest the next day. At the meeting, the patients signed an informed consent form, and they were further screened for eligibility and allocated to an experimental or sham training group"

4a-iii) Information giving during recruitment

Information given during recruitment. Specify how participants were briefed for recruitment and in the informed consent procedures (e.g., publish the informed consent documentation as appendix, see also item X26), as this information may have an effect on user self-selection, user expectation and may also bias results.

	1	2	3	4	5	
subitem not at all important	○	○	○	○	◉	essential

Clear selection

Does your paper address subitem 4a-iii?

Copy and paste relevant sections from the manuscript (include quotes in quotation marks "like this" to indicate direct quotes from your manuscript), or elaborate on this item by providing additional information not in the ms, or briefly explain why the item is not applicable/relevant for your study

"Patients who expressed interest in participating were referred to a research assistant who provided verbal and written information about the study following a standardized procedure."

4b) Settings and locations where the data were collected

Does your paper address CONSORT subitem 4b? *

Copy and paste relevant sections from the manuscript (include quotes in quotation marks "like this" to indicate direct quotes from your manuscript), or elaborate on this item by providing additional information not in the ms, or briefly explain why the item is not applicable/relevant for your study

"Patients from the publicly financed, outpatient alcohol treatment clinic in Odense, Denmark were consecutively invited to take part in the study until a final sample of 72 patients was obtained. The recruitment took place between September 2021 and September 2022."

4b-i) Report if outcomes were (self-)assessed through online questionnaires

Clearly report if outcomes were (self-)assessed through online questionnaires (as common in web-based trials) or otherwise.

	1	2	3	4	5	
subitem not at all important	(●)	( )	( )	( )	( )	essential

Clear selection

Does your paper address subitem 4b-i? *

Copy and paste relevant sections from the manuscript (include quotes in quotation marks "like this" to indicate direct quotes from your manuscript), or elaborate on this item by providing additional information not in the ms, or briefly explain why the item is not applicable/relevant for your study

All questionnaires were completed by the help of a research assistant, so no questionnaires were sent out for the patients to fill out on their own.

4b-ii) Report how institutional affiliations are displayed

Report how institutional affiliations are displayed to potential participants [on ehealth media], as affiliations with prestigious hospitals or universities may affect volunteer rates, use, and reactions with regards to an intervention.(Not a required item – describe only if this may bias results)

	1	2	3	4	5	
subitem not at all important	(selected)	○	○	○	○	essential

Clear selection

Does your paper address subitem 4b-ii?

Copy and paste relevant sections from the manuscript (include quotes in quotation marks "like this" to indicate direct quotes from your manuscript), or elaborate on this item by providing additional information not in the ms, or briefly explain why the item is not applicable/relevant for your study

Recruited from an alcohol outpatient treatment facility in Odense

5) The interventions for each group with sufficient details to allow replication, including how and when they were actually administered

5-i) Mention names, credential, affiliations of the developers, sponsors, and owners

Mention names, credential, affiliations of the developers, sponsors, and owners [6] (if authors/evaluators are owners or developer of the software, this needs to be declared in a “Conflict of interest” section or mentioned elsewhere in the manuscript).

	1	2	3	4	5	
subitem not at all important	○	○	○	○	◉	essential

Clear selection

Does your paper address subitem 5-i?

Copy and paste relevant sections from the manuscript (include quotes in quotation marks "like this" to indicate direct quotes from your manuscript), or elaborate on this item by providing additional information not in the ms, or briefly explain why the item is not applicable/relevant for your study

"The cognitive training application used in this study was adapted from the publicly available Brain+ Recover application 48, which was developed by neuropsycholo-gists at the Centre for Traumatic Brain Injury, University of Copenhagen in collabo-ration with the company Brain+. Multiple versions of the Brain+ Recover applica-tion have been tested by other research groups 49-51. For the present trial, the Brain+ Recover application was adapted, a sham-version was created, and the two adapted versions were renamed Brain+ Alco-Recover."

5-ii) Describe the history/development process

Describe the history/development process of the application and previous formative evaluations (e.g., focus groups, usability testing), as these will have an impact on adoption/use rates and help with interpreting results.

	1	2	3	4	5	
subitem not at all important	◉	○	○	○	○	essential

Clear selection

Does your paper address subitem 5-ii?

Copy and paste relevant sections from the manuscript (include quotes in quotation marks "like this" to indicate direct quotes from your manuscript), or elaborate on this item by providing additional information not in the ms, or briefly explain why the item is not applicable/relevant for your study

In addition to our trial being a pilot and feasibility study, our app was based on a publicly available application, which was adapted to an alcohol population.

5-iii) Revisions and updating

Revisions and updating. Clearly mention the date and/or version number of the application/intervention (and comparator, if applicable) evaluated, or describe whether the intervention underwent major changes during the evaluation process, or whether the development and/or content was "frozen" during the trial. Describe dynamic components such as news feeds or changing content which may have an impact on the replicability of the intervention (for unexpected events see item 3b).

	1	2	3	4	5	
subitem not at all important	○	○	◉	○	○	essential

Clear selection

Does your paper address subitem 5-iii?

Copy and paste relevant sections from the manuscript (include quotes in quotation marks "like this" to indicate direct quotes from your manuscript), or elaborate on this item by providing additional information not in the ms, or briefly explain why the item is not applicable/relevant for your study

"The developer team at Brain+ ensured that the training program in both the adapted versions consisted of the various types of games targeting multiple cogni-tive domains. To test the effects of the training program with high ecological validi-ty, the Brain+ Alco-Recover application was used by the patients at home without any guidance from the researchers."

Our app was in its early development phases, and testet for its feasibility. The application did not undergo any changes or adaptations during the recruitment of patients.

5-iv) Quality assurance methods

Provide information on quality assurance methods to ensure accuracy and quality of information provided [1], if applicable.

	1	2	3	4	5	
subitem not at all important	(●)	○	○	○	○	essential

Clear selection

Does your paper address subitem 5-iv?

Copy and paste relevant sections from the manuscript (include quotes in quotation marks "like this" to indicate direct quotes from your manuscript), or elaborate on this item by providing additional information not in the ms, or briefly explain why the item is not applicable/relevant for your study

Our assessment battery was brief and based on standardised measures. All data was collected by trained research assistants, who ensured accurate data collection during the entire study

5-v) Ensure replicability by publishing the source code, and/or providing screenshots/screen-capture video, and/or providing flowcharts of the algorithms used

Ensure replicability by publishing the source code, and/or providing screenshots/screen-capture video, and/or providing flowcharts of the algorithms used. Replicability (i.e., other researchers should in principle be able to replicate the study) is a hallmark of scientific reporting.

	1	2	3	4	5	
subitem not at all important	(selected)	○	○	○	○	essential

Clear selection

Does your paper address subitem 5-v?

Copy and paste relevant sections from the manuscript (include quotes in quotation marks "like this" to indicate direct quotes from your manuscript), or elaborate on this item by providing additional information not in the ms, or briefly explain why the item is not applicable/relevant for your study

The application itself is publicly available. We do not have the rights to share any source code to the program, as these belong to the company Brain+, who are not willing to share confidential information on the source code.

5-vi) Digital preservation

Digital preservation: Provide the URL of the application, but as the intervention is likely to change or disappear over the course of the years; also make sure the intervention is archived (Internet Archive, webcitation.org, and/or publishing the source code or screenshots/videos alongside the article). As pages behind login screens cannot be archived, consider creating demo pages which are accessible without login.

	1	2	3	4	5	
subitem not at all important	○	○	○	○	◉	essential

Clear selection

Does your paper address subitem 5-vi?

Copy and paste relevant sections from the manuscript (include quotes in quotation marks "like this" to indicate direct quotes from your manuscript), or elaborate on this item by providing additional information not in the ms, or briefly explain why the item is not applicable/relevant for your study

We have provided screenshots of the games used in the training program as a multimedia appendix

5-vii) Access

Access: Describe how participants accessed the application, in what setting/context, if they had to pay (or were paid) or not, whether they had to be a member of specific group. If known, describe how participants obtained “access to the platform and Internet” [1]. To ensure access for editors/reviewers/readers, consider to provide a “backdoor” login account or demo mode for reviewers/readers to explore the application (also important for archiving purposes, see vi).

	1	2	3	4	5	
subitem not at all important	○	○	○	○	◉	essential

Clear selection

Does your paper address subitem 5-vii? *

Copy and paste relevant sections from the manuscript (include quotes in quotation marks "like this" to indicate direct quotes from your manuscript), or elaborate on this item by providing additional information not in the ms, or briefly explain why the item is not applicable/relevant for your study

"Lastly, the research assistant assisted the patients with installing the application on their smartphone/tablet and provided instructions on how to use it (see section on “Instruction for the experimental and sham group”)."

5-viii) Mode of delivery, features/functionalities/components of the intervention and comparator, and the theoretical framework

Describe mode of delivery, features/functionalities/components of the intervention and comparator, and the theoretical framework [6] used to design them (instructional strategy [1], behaviour change techniques, persuasive features, etc., see e.g., [7, 8] for terminology). This includes an in-depth description of the content (including where it is coming from and who developed it) [1],” whether [and how] it is tailored to individual circumstances and allows users to track their progress and receive feedback” [6]. This also includes a description of communication delivery channels and – if computer-mediated communication is a component – whether communication was synchronous or asynchronous [6]. It also includes information on presentation strategies [1], including page design principles, average amount of text on pages, presence of hyperlinks to other resources, etc. [1].

	1	2	3	4	5	
subitem not at all important	○	○	○	○	●	essential

Clear selection

Does your paper address subitem 5-viii? *

Copy and paste relevant sections from the manuscript (include quotes in quotation marks "like this" to indicate direct quotes from your manuscript), or elaborate on this item by providing additional information not in the ms, or briefly explain why the item is not applicable/relevant for your study

"The experimental version of Brain+ Alco-Recover consisted of a personalized training program initially created from the pre-assessment, and the difficulty level was adjusted to fit the patient's performance level measured during the pre-assessment. In this version, each time the patient successfully completed a game session, the results were visually presented, and the level of difficulty increased slightly to match the patient's current performance level. These incremental changes in difficulty included lower exposure time, additional items to recall, or more obstacles in the game, which would scale proportionally according to the pa-tient's performance level. If the level of difficulty became too challenging and the patient could no longer complete the game session, the level of difficulty would de-crease accordingly.
The personalized, daily training sessions consisted of the six different games, which were combined in two blocks of four games each. The training blocks alter-nated between the selection of games, but all the games appeared with the same frequency. The in-game algorithm ensured that the two blocks were personalized in terms of the duration of each game, and the cognitive domains in which the pa-tient underperformed in during the pre-assessment were thus targeted for more intensive training. Each game took on average two minutes and 40 seconds to complete, but one training block lasted 10 minutes."

5-ix) Describe use parameters

Describe use parameters (e.g., intended "doses" and optimal timing for use). Clarify what instructions or recommendations were given to the user, e.g., regarding timing, frequency, heaviness of use, if any, or was the intervention used ad libitum.

	1	2	3	4	5	
subitem not at all important	○	○	○	○	◉	essential

Clear selection

Does your paper address subitem 5-ix?

Copy and paste relevant sections from the manuscript (include quotes in quotation marks "like this" to indicate direct quotes from your manuscript), or elaborate on this item by providing additional information not in the ms, or briefly explain why the item is not applicable/relevant for your study

"Based on other studies that examined a similar cognitive training program 21,23,27, patients in both the experimental and sham groups were instructed to use the ap-plication for 20 minutes a day, five days a week, for one month (i.e., a minimum of 400 minutes or six hours and 40 minutes of total in-game time for the entire inter-vention). Thus, the patients in both groups had to complete a training session con-sisting of two blocks lasting 10 minutes each (i.e., two blocks of four games each) to fulfill the minimum requirements for a daily training session. Adhering to this pre-defined training program would result in the completion of 20 training sessions (i.e., 40 training blocks) and this was equal to 150 games, which meant that each of the six different games appeared 25 times."

5-x) Clarify the level of human involvement

Clarify the level of human involvement (care providers or health professionals, also technical assistance) in the e-intervention or as co-intervention (detail number and expertise of professionals involved, if any, as well as "type of assistance offered, the timing and frequency of the support, how it is initiated, and the medium by which the assistance is delivered". It may be necessary to distinguish between the level of human involvement required for the trial, and the level of human involvement required for a routine application outside of a RCT setting (discuss under item 21 – generalizability).

	1	2	3	4	5	
subitem not at all important	○	○	○	○	◉	essential

Clear selection

Does your paper address subitem 5-x?

Copy and paste relevant sections from the manuscript (include quotes in quotation marks "like this" to indicate direct quotes from your manuscript), or elaborate on this item by providing additional information not in the ms, or briefly explain why the item is not applicable/relevant for your study

"To test the effects of the training program with high ecological validity, the Brain+ Alco-Recover application was used by the patients at home without any guidance from the researchers."

5-xi) Report any prompts/reminders used

Report any prompts/reminders used: Clarify if there were prompts (letters, emails, phone calls, SMS) to use the application, what triggered them, frequency etc. It may be necessary to distinguish between the level of prompts/reminders required for the trial, and the level of prompts/reminders for a routine application outside of a RCT setting (discuss under item 21 – generalizability).

	1	2	3	4	5	
subitem not at all important	◉	○	○	○	○	essential

Clear selection

Does your paper address subitem 5-xi? *

Copy and paste relevant sections from the manuscript (include quotes in quotation marks "like this" to indicate direct quotes from your manuscript), or elaborate on this item by providing additional information not in the ms, or briefly explain why the item is not applicable/relevant for your study

This was an early edition of the training application where prompts and notification were not yet incorporated. Also, this study testet the usage of the training application in an unguided setting, meaning that the patients were not assisted or reminded of their daily training sessions.

5-xii) Describe any co-interventions (incl. training/support)

Describe any co-interventions (incl. training/support): Clearly state any interventions that are provided in addition to the targeted eHealth intervention, as ehealth intervention may not be designed as stand-alone intervention. This includes training sessions and support [1]. It may be necessary to distinguish between the level of training required for the trial, and the level of training for a routine application outside of a RCT setting (discuss under item 21 – generalizability.

	1	2	3	4	5	
subitem not at all important	(●)	○	○	○	○	essential

Clear selection

Does your paper address subitem 5-xii? *

Copy and paste relevant sections from the manuscript (include quotes in quotation marks "like this" to indicate direct quotes from your manuscript), or elaborate on this item by providing additional information not in the ms, or briefly explain why the item is not applicable/relevant for your study

This was delivered as add-on to the conventional treatment at the outpatient facility, so no other interventions were delivered.

6a) Completely defined pre-specified primary and secondary outcome measures, including how and when they were assessed

Does your paper address CONSORT subitem 6a? *

Copy and paste relevant sections from the manuscript (include quotes in quotation marks "like this" to indicate direct quotes from your manuscript), or elaborate on this item by providing additional information not in the ms, or briefly explain why the item is not applicable/relevant for your study

"Cognitive performance was measured with assessment versions of the various training games in Brain+ Alco-Recover. Since many of the games tapped into over-lapping cognitive processes, the games were re-grouped across their pre-defined in-game categories. For the present study, the in-game category of memory was also split up so that working memory as well as learning and episodic memory could be assessed separately. The performance in four cognitive domains were pre-specified as executive functions (i.e., the in-game Pathfinder), processing speed and attention (i.e., the in-games Perception Speed and Attention Island), working memory (i.e., the in-games Memory Lane and Bulky Codes), and finally learning and episodic memory (i.e., the in-game Remember Me).
Bugs in the Brain+ application as well as difficulties with the implementation of a sham-version of the games for executive functions and episodic memory meant that the performance in these domains could not be assessed for the sham group. Thus, the executive and episodic memory domains were omitted, and the primary outcomes reported in this study was on two domains: processing speed/attention and working memory."

"Secondary outcomes: Alcohol consumption was measured using the following two items derived from the alcohol module in the ASI: 1) days with any alcohol consumption in the past 30 days, and 2) days with excessive drinking (i.e., three units or more) in the past 30 days 46. Average craving level and highest craving level during the past week and the past 30 days was measured using the visual analogue scale (VAS) 47."

6a-i) Online questionnaires: describe if they were validated for online use and apply CHERRIES items to describe how the questionnaires were designed/deployed

If outcomes were obtained through online questionnaires, describe if they were validated for online use and apply CHERRIES items to describe how the questionnaires were designed/deployed [9].

	1	2	3	4	5	
subitem not at all important	◉	○	○	○	○	essential

Clear selection

Does your paper address subitem 6a-i?

Copy and paste relevant sections from manuscript text

No online questionnaires were delivered to the patients in this study

6a-ii) Describe whether and how "use" (including intensity of use/dosage) was defined/measured/monitored

Describe whether and how "use" (including intensity of use/dosage) was defined/measured/monitored (logins, logfile analysis, etc.). Use/adoption metrics are important process outcomes that should be reported in any ehealth trial.

	1	2	3	4	5	
subitem not at all important	○	○	○	○	◉	essential

Clear selection

Does your paper address subitem 6a-ii?

Copy and paste relevant sections from manuscript text

"The process outcomes were defined as the actual usage of the individual games in the training program For the experimental group, a minimum requirement of five completed game trials for each of the six games (i.e., four completed training sessions or 30 completed games in total) was deemed as acceptable and this was equal to approximately 80 minutes of total training time (i.e., 20% of the instructed duration for the pre-defined training program)."

6a-iii) Describe whether, how, and when qualitative feedback from participants was obtained

Describe whether, how, and when qualitative feedback from participants was obtained (e.g., through emails, feedback forms, interviews, focus groups).

	1	2	3	4	5	
subitem not at all important	(●)	( )	( )	( )	( )	essential

Clear selection

Does your paper address subitem 6a-iii?

Copy and paste relevant sections from manuscript text

Qualitative feedback was not collected for this pilot and feasibility trial

6b) Any changes to trial outcomes after the trial commenced, with reasons

Does your paper address CONSORT subitem 6b? *

Copy and paste relevant sections from the manuscript (include quotes in quotation marks "like this" to indicate direct quotes from your manuscript), or elaborate on this item by providing additional information not in the ms, or briefly explain why the item is not applicable/relevant for your study

"Although the error in the game Pathfinder and the lack of a sham version of the game Remember Me meant that these games could not be used as assessments for executive functions and episodic memory, respectively, the number of completed game trials for both games was still logged and used as an outcome for the acceptability of the entire training program."

7a) How sample size was determined

NPT: When applicable, details of whether and how the clustering by care provides or centers was addressed

7a-i) Describe whether and how expected attrition was taken into account when calculating the sample size

Describe whether and how expected attrition was taken into account when calculating the sample size.

	1	2	3	4	5	
subitem not at all important	○	○	○	○	◉	essential

Clear selection

Does your paper address subitem 7a-i?

Copy and paste relevant sections from manuscript title (include quotes in quotation marks "like this" to indicate direct quotes from your manuscript), or elaborate on this item by providing additional information not in the ms, or briefly explain why the item is not applicable/relevant for your study

"Although the present study was only a pilot trial without a formal power calcula-tion, the goal was to include a minimum of 60 patients equally distributed across the two groups to approach a normal distribution in accordance with the central limit theorem. Based on prior trials that examined cognitive training 23,27,43, we ex-pected an attrition rate of 20% (n =12 patients). Thus, 12 additional patients were recruited to ensure that we obtained the required sample size of 30 patients in each group."

7b) When applicable, explanation of any interim analyses and stopping guidelines

Does your paper address CONSORT subitem 7b? *

Copy and paste relevant sections from the manuscript (include quotes in quotation marks "like this" to indicate direct quotes from your manuscript), or elaborate on this item by providing additional information not in the ms, or briefly explain why the item is not applicable/relevant for your study

This was a pilot and feasibility study, so we did not conduct any interim analyses.

8a) Method used to generate the random allocation sequence

NPT: When applicable, how care providers were allocated to each trial group

Does your paper address CONSORT subitem 8a? *

Copy and paste relevant sections from the manuscript (include quotes in quotation marks "like this" to indicate direct quotes from your manuscript), or elaborate on this item by providing additional information not in the ms, or briefly explain why the item is not applicable/relevant for your study

"An urn randomization technique was used without any stratification 42, and the al-location of patients to the groups was performed by an independent statistician."

8b) Type of randomisation; details of any restriction (such as blocking and block size)

Does your paper address CONSORT subitem 8b? *

Copy and paste relevant sections from the manuscript (include quotes in quotation marks "like this" to indicate direct quotes from your manuscript), or elaborate on this item by providing additional information not in the ms, or briefly explain why the item is not applicable/relevant for your study

"An urn randomization technique was used without any stratification 42, and the al-location of patients to the groups was performed by an independent statistician. Although the present study was only a pilot trial without a formal power calculation, the goal was to include a minimum of 60 patients equally distributed across the two groups to approach a normal distribution in accordance with the central limit theorem."

9) Mechanism used to implement the random allocation sequence (such as sequentially numbered containers), describing any steps taken to conceal the sequence until interventions were assigned

Does your paper address CONSORT subitem 9? *

Copy and paste relevant sections from the manuscript (include quotes in quotation marks "like this" to indicate direct quotes from your manuscript), or elaborate on this item by providing additional information not in the ms, or briefly explain why the item is not applicable/relevant for your study

"The patients were blinded to their allocated group, and the research assistants who performed the randomization were also unaware of which version of the training application they helped to install on the patients' smartphone/tablet. This was achieved by assigning a random number to the two versions of the training application, and these two designated numbers were only known to an independent researcher who did not take part in the assessment of the patients."

10) Who generated the random allocation sequence, who enrolled participants, and who assigned participants to interventions

Does your paper address CONSORT subitem 10? *

Copy and paste relevant sections from the manuscript (include quotes in quotation marks "like this" to indicate direct quotes from your manuscript), or elaborate on this item by providing additional information not in the ms, or briefly explain why the item is not applicable/relevant for your study

"An urn randomization technique was used without any stratification 42, and the allocation of patients to the groups was performed by an independent statistician."

11a) If done, who was blinded after assignment to interventions (for example, participants, care providers, those assessing outcomes) and how

NPT: Whether or not administering co-interventions were blinded to group assignment

11a-i) Specify who was blinded, and who wasn't

Specify who was blinded, and who wasn't. Usually, in web-based trials it is not possible to blind the participants [1, 3] (this should be clearly acknowledged), but it may be possible to blind outcome assessors, those doing data analysis or those administering co-interventions (if any).

	1	2	3	4	5	
subitem not at all important	○	○	○	○	◉	essential

Clear selection

Does your paper address subitem 11a-i? *

Copy and paste relevant sections from the manuscript (include quotes in quotation marks "like this" to indicate direct quotes from your manuscript), or elaborate on this item by providing additional information not in the ms, or briefly explain why the item is not applicable/relevant for your study

"The patients were blinded to their allocated group, and the research assistants who performed the randomization were also unaware of which version of the training application they helped to install on the patients' smartphone/tablet."

11a-ii) Discuss e.g., whether participants knew which intervention was the "intervention of interest" and which one was the "comparator"

Informed consent procedures (4a-ii) can create biases and certain expectations - discuss e.g., whether participants knew which intervention was the "intervention of interest" and which one was the "comparator".

	1	2	3	4	5	
subitem not at all important	○	○	○	○	◉	essential

Clear selection

Does your paper address subitem 11a-ii?

Copy and paste relevant sections from the manuscript (include quotes in quotation marks "like this" to indicate direct quotes from your manuscript), or elaborate on this item by providing additional information not in the ms, or briefly explain why the item is not applicable/relevant for your study

"The patients were blinded to their allocated group, and the research assistants who performed the randomization were also unaware of which version of the training application they helped to install on the patients' smartphone/tablet. This was achieved by assigning a random number to the two versions of the training application, and these two designated numbers were only known to an independ-ent researcher who did not take part in the assessment of the patients."

11b) If relevant, description of the similarity of interventions

(this item is usually not relevant for ehealth trials as it refers to similarity of a placebo or sham intervention to a active medication/intervention)

Does your paper address CONSORT subitem 11b? *

Copy and paste relevant sections from the manuscript (include quotes in quotation marks "like this" to indicate direct quotes from your manuscript), or elaborate on this item by providing additional information not in the ms, or briefly explain why the item is not applicable/relevant for your study

The experimental and sham version were identical, and the true meaning of the two versions were only known by an independent researcher

12a) Statistical methods used to compare groups for primary and secondary outcomes

NPT: When applicable, details of whether and how the clustering by care providers or centers was addressed

Does your paper address CONSORT subitem 12a? *

Copy and paste relevant sections from the manuscript (include quotes in quotation marks "like this" to indicate direct quotes from your manuscript), or elaborate on this item by providing additional information not in the ms, or briefly explain why the item is not applicable/relevant for your study

"The baseline characteristics of patients in both study arms were summarized using means and standard deviations for continuous variables, and frequencies and per-centages for categorical variables. Differences between the two arms were assessed using independent samples t-tests for continuous variables, Chi-square tests for categorical variables, and the Mann-Whitney U test for non-normally distributed continuous variables."

12a-i) Imputation techniques to deal with attrition / missing values

Imputation techniques to deal with attrition / missing values: Not all participants will use the intervention/comparator as intended and attrition is typically high in ehealth trials. Specify how participants who did not use the application or dropped out from the trial were treated in the statistical analysis (a complete case analysis is strongly discouraged, and simple imputation techniques such as LOCF may also be problematic [4]).

	1	2	3	4	5	
subitem not at all important	○	○	○	○	●	essential

Clear selection

Does your paper address subitem 12a-i? *

Copy and paste relevant sections from the manuscript (include quotes in quotation marks "like this" to indicate direct quotes from your manuscript), or elaborate on this item by providing additional information not in the ms, or briefly explain why the item is not applicable/relevant for your study

"The analyses of primary outcomes were conducted as both intention-to-treat and as per-protocol (i.e., the minimum acceptable number of five completed game trials for each game), and the secondary outcomes as well as the feasibility and acceptability outcome were conducted as intention-to-treat only."

12b) Methods for additional analyses, such as subgroup analyses and adjusted analyses

Does your paper address CONSORT subitem 12b? *

Copy and paste relevant sections from the manuscript (include quotes in quotation marks "like this" to indicate direct quotes from your manuscript), or elaborate on this item by providing additional information not in the ms, or briefly explain why the item is not applicable/relevant for your study

"The primary outcomes of the three cognitive domains as well as the brain score for the individual cognitive games were evaluated with independent samples t-tests by analyzing the differences in mean change between the baseline assess-ment and the assessment during the patients' last training session. The secondary clinical outcomes were evaluated with linear mixed models. These models incorpo-rated data collected at baseline, post-treatment, and at 6-months follow-up for the secondary outcome measures, which included number of drinking days (DD), number of heavy drinking days (HDD), mean craving level (MCL), and highest craving level (HCL). The linear mixed models used the patient number as a ran-dom effect to account for within-subject correlation and time (i.e., DD, HDD, MCL, HCL at T0, T1, and at T2; cognitive assessment at T0 and T1). The model also in-cluded a time by randomization arm interaction as fixed effects to evaluate differ-ential changes over time between the experimental and the sham group."

X26) REB/IRB Approval and Ethical Considerations [recommended as subheading under "Methods"] (not a CONSORT item)

X26-i) Comment on ethics committee approval

	1	2	3	4	5	
subitem not at all important	○	○	○	○	◉	essential

Clear selection

Does your paper address subitem X26-i?

Copy and paste relevant sections from the manuscript (include quotes in quotation marks "like this" to indicate direct quotes from your manuscript), or elaborate on this item by providing additional information not in the ms, or briefly explain why the item is not applicable/relevant for your study

"The present study was approved by the Research Ethics Committee for the Region of Southern Denmark (Project ID: S-20200199), and it was conducted in accord-ance with the Declaration of Helsinki 39"

x26-ii) Outline informed consent procedures

Outline informed consent procedures e.g., if consent was obtained offline or online (how? Checkbox, etc.?), and what information was provided (see 4a-ii). See [6] for some items to be included in informed consent documents.

	1	2	3	4	5	
subitem not at all important	○	○	○	○	◉	essential

Clear selection

Does your paper address subitem X26-ii?

Copy and paste relevant sections from the manuscript (include quotes in quotation marks "like this" to indicate direct quotes from your manuscript), or elaborate on this item by providing additional information not in the ms, or briefly explain why the item is not applicable/relevant for your study

"The research assistant scheduled a meeting with those patients who agreed to par-ticipate, which took place at the earliest the next day. At the meeting, the patients signed an informed consent form, and they were further screened for eligibility and allocated to an experimental or sham training group (see section on “Allocation and blinding”)."

X26-iii) Safety and security procedures

Safety and security procedures, incl. privacy considerations, and any steps taken to reduce the likelihood or detection of harm (e.g., education and training, availability of a hotline)

	1	2	3	4	5	
subitem not at all important	◉	○	○	○	○	essential

Clear selection

Does your paper address subitem X26-iii?

Copy and paste relevant sections from the manuscript (include quotes in quotation marks "like this" to indicate direct quotes from your manuscript), or elaborate on this item by providing additional information not in the ms, or briefly explain why the item is not applicable/relevant for your study

A publicly available application that was not evaluated as posing any harm or adverse effects for the present trial.

RESULTS

13a) For each group, the numbers of participants who were randomly assigned, received intended treatment, and were analysed for the primary outcome

NPT: The number of care providers or centers performing the intervention in each group and the number of patients treated by each care provider in each center

Does your paper address CONSORT subitem 13a? *

Copy and paste relevant sections from the manuscript (include quotes in quotation marks "like this" to indicate direct quotes from your manuscript), or elaborate on this item by providing additional information not in the ms, or briefly explain why the item is not applicable/relevant for your study

"A total of 72 patients were randomized to either the experimental group (n = 36) or the sham group (n = 36)."

13b) For each group, losses and exclusions after randomisation, together with reasons

Does your paper address CONSORT subitem 13b? (NOTE: Preferably, this is shown in a CONSORT flow diagram) *

Copy and paste relevant sections from the manuscript (include quotes in quotation marks "like this" to indicate direct quotes from your manuscript), or elaborate on this item by providing additional information not in the ms, or briefly explain why the item is not applicable/relevant for your study

"One patient in the experimental group withdrew consent to participate after the baseline assessment and thus data for this person were deleted (see Figure 1). Fif-ty patients completed the post-treatment assessment (i.e., experimental group: n = 29; sham group: n = 21), and 43 patients completed the 6-months follow-up assessment (i.e., experimental group: n = 25; sham group: n = 18)."

13b-i) Attrition diagram

Strongly recommended: An attrition diagram (e.g., proportion of participants still logging in or using the intervention/comparator in each group plotted over time, similar to a survival curve) or other figures or tables demonstrating usage/dose/engagement.

	1	2	3	4	5	
subitem not at all important	○	○	○	○	◉	essential

Clear selection

Does your paper address subitem 13b-i?

Copy and paste relevant sections from the manuscript or cite the figure number if applicable (include quotes in quotation marks "like this" to indicate direct quotes from your manuscript), or elaborate on this item by providing additional information not in the ms, or briefly explain why the item is not applicable/relevant for your study

"One patient in the experimental group withdrew consent to participate after the baseline assessment and thus data for this person were deleted (see Figure 1). Fif-ty patients completed the post-treatment assessment (i.e., experimental group: n = 29; sham group: n = 21), and 43 patients completed the 6-months follow-up as-sessment (i.e., experimental group: n = 25; sham group: n = 18)."

ALSO, SEE FLOW CHART IN FIGURE 1

14a) Dates defining the periods of recruitment and follow-up

Does your paper address CONSORT subitem 14a? *

Copy and paste relevant sections from the manuscript (include quotes in quotation marks "like this" to indicate direct quotes from your manuscript), or elaborate on this item by providing additional information not in the ms, or briefly explain why the item is not applicable/relevant for your study

"The recruitment took place between September 2021 and September 2022."

14a-i) Indicate if critical “secular events” fell into the study period

Indicate if critical “secular events” fell into the study period, e.g., significant changes in Internet resources available or “changes in computer hardware or Internet delivery resources”

	1	2	3	4	5	
subitem not at all important	◉	○	○	○	○	essential

Clear selection

Does your paper address subitem 14a-i?

Copy and paste relevant sections from the manuscript (include quotes in quotation marks "like this" to indicate direct quotes from your manuscript), or elaborate on this item by providing additional information not in the ms, or briefly explain why the item is not applicable/relevant for your study

We did not have any changes to the training application, while the study was still ongoing.

14b) Why the trial ended or was stopped (early)

Does your paper address CONSORT subitem 14b? *

Copy and paste relevant sections from the manuscript (include quotes in quotation marks "like this" to indicate direct quotes from your manuscript), or elaborate on this item by providing additional information not in the ms, or briefly explain why the item is not applicable/relevant for your study

"The recruitment took place between September 2021 and September 2022."

15) A table showing baseline demographic and clinical characteristics for each group

NPT: When applicable, a description of care providers (case volume, qualification, expertise, etc.) and centers (volume) in each group

Does your paper address CONSORT subitem 15? *

Copy and paste relevant sections from the manuscript (include quotes in quotation marks "like this" to indicate direct quotes from your manuscript), or elaborate on this item by providing additional information not in the ms, or briefly explain why the item is not applicable/relevant for your study

YES, SEE TABLE 1

ALSO

"No significant differences were found between the experimental and sham groups regarding age, gender, education, employment, and clinical variables (see Table 1). More than half of the patients were male (74.6%; n = 53), and the overall mean age of the patients was 43.3 years (SD = 10.9). Three of the included patients were 64, 67, and 70 years old, hence deviating from the eligibility criteria specified in the pre-registered protocol. Concerning the clinical variables, over 90% of the patients in both groups had an AUD (i.e., F10.2 diagnosis of alcohol dependence in the ICD-10), while the remaining patients fulfilled the criteria for harmful use of alcohol (i.e., F10.1 diagnosis in the ICD-10). Approximately 60% of the patients in both groups reported having previously received outpatient treatment for alcohol misuse. Previous inpatient treatment for alcohol misuse was reported less frequently, with only 9% of the patients in the experimental group and 8% in the sham group reporting this. The age of onset of heavy drinking was approximately 28 years in both groups."

15-i) Report demographics associated with digital divide issues

In ehealth trials it is particularly important to report demographics associated with digital divide issues, such as age, education, gender, social-economic status, computer/Internet/ehealth literacy of the participants, if known.

	1	2	3	4	5	
subitem not at all important	○	○	○	○	◉	essential

Clear selection

Does your paper address subitem 15-i? *

Copy and paste relevant sections from the manuscript (include quotes in quotation marks "like this" to indicate direct quotes from your manuscript), or elaborate on this item by providing additional information not in the ms, or briefly explain why the item is not applicable/relevant for your study

"No significant differences were found between the experimental and sham groups regarding age, gender, education, employment, and clinical variables (see Table 1). More than half of the patients were male (74.6%; n = 53), and the overall mean age of the patients was 43.3 years (SD = 10.9). Three of the included patients were 64, 67, and 70 years old, hence deviating from the eligibility criteria specified in the pre-registered protocol. Concerning the clinical variables, over 90% of the patients in both groups had an AUD (i.e., F10.2 diagnosis of alcohol dependence in the ICD-10), while the remaining patients fulfilled the criteria for harmful use of alcohol (i.e., F10.1 diagnosis in the ICD-10). Approximately 60% of the patients in both groups reported having previously received outpatient treatment for alcohol misuse. Previous inpatient treatment for alcohol misuse was reported less frequently, with only 9% of the patients in the experimental group and 8% in the sham group reporting this. The age of onset of heavy drinking was approximately 28 years in both groups."

16) For each group, number of participants (denominator) included in each analysis and whether the analysis was by original assigned groups

16-i) Report multiple “denominators” and provide definitions

Report multiple “denominators” and provide definitions: Report N’s (and effect sizes) “across a range of study participation [and use] thresholds” [1], e.g., N exposed, N consented, N used more than x times, N used more than y weeks, N participants “used” the intervention/comparator at specific pre-defined time points of interest (in absolute and relative numbers per group). Always clearly define “use” of the intervention.

	1	2	3	4	5	
subitem not at all important	○	○	○	○	●	essential

Clear selection

Does your paper address subitem 16-i? *

Copy and paste relevant sections from the manuscript (include quotes in quotation marks "like this" to indicate direct quotes from your manuscript), or elaborate on this item by providing additional information not in the ms, or briefly explain why the item is not applicable/relevant for your study

"The cognitive assessment revealed significant differences between the experi-mental and sham groups at baseline (see Table 1) in the domain for processing speed and attention as well as the domain for working memory (ps < .001). The experimental group performed better in the domain for processing speed and at-tention (M = 440.6, SD = 166.3; n = 17) compared to the sham group (M = 70.4, SD = 70.5; n = 22). Further, the average scores on the two individual games were significantly higher in the experimental group (Attention Island: M = 86.3, SD = 71.8; n = 13 and Speed Perception: M = 650.1, SD = 152.1; n = 17) compared to the sham group (Attention Island: M = 12.3, SD = 4.3; n = 22 and Speed Perception: M = 208.6, SD = 134.5; n = 13). The experimental group performed better in the do-main for working memory (M = 210.3, SD = 36.8; n = 23) compared to the sham group (M = 127.4, SD = 7.7; n = 23). Moreover, the average scores on the two individual games were significantly higher in the experimental group (Bulky Codes: M = 212.3, SD = 39.8; n = 18 and Memory Lane: M = 204.8, SD = 44.8; n = 23) com-pared to the sham group (Bulky Codes: M = 137.2, SD = 10.7; n = 17; Memory Lane: M = 121.9, SD = 6.1; n = 22)."

"The first criterion specifying that more than half of the patients had to fulfill the minimum requirement of five completed game trials for each of the games was reached in five out of the six games in the experimental version of the Brain+ Alco-Recover application (see supplementary material, Table S1). [...] The second criterion specifying that more than two-thirds of the patients had to complete a minimum of 30 game trials regardless of game type was reached in both groups. In the experimental group, 74.3 % of the patients (n = 26) completed a minimum of 30 game trials, and the corresponding percentage in the sham group was 66.7 % (n = 24). The average number of game trials completed by the patients fulfilling this criterion was 217.4 trials in the experimental group (i.e., approximately a total of 577 minutes of training time) and 345.6 trials in the sham group (i.e., approximately a total of 919 minutes of training time)."

ALSO SEE TABLE 1 ON BASELINE CHARACTERISTICS

16-ii) Primary analysis should be intent-to-treat

Primary analysis should be intent-to-treat, secondary analyses could include comparing only “users”, with the appropriate caveats that this is no longer a randomized sample (see 18-i).

	1	2	3	4	5	
subitem not at all important	○	○	○	○	◉	essential

Clear selection

Does your paper address subitem 16-ii?

Copy and paste relevant sections from the manuscript (include quotes in quotation marks "like this" to indicate direct quotes from your manuscript), or elaborate on this item by providing additional information not in the ms, or briefly explain why the item is not applicable/relevant for your study

"Comparisons between the baseline and post-treatment assessments (see figure 2) showed significant differences between the two groups in the domain for pro-cessing speed and attention (P = .019) as well as the domain for working memory (P < .001). Closer inspection revealed that the differences were driven by higher mean change scores in the experimental group (processing speed and attention: M = 9.93, 95% CI [-61.2; 81.0] and working memory: M = 134.8, 95% CI [102.2; 167.5]) compared to the sham group (processing speed and attention: M = -72.8, 95% CI[-100.1; -45.6]; and working memory (M = 38.6, 95% CI [22.8; 54.4]). Re-garding the individual games in these domains, significant differences were only present for Bulky Codes (P < .001) and Memory Lane (P < .001), which were driv-en by improvements in the experimental group (i.e., Bulky Codes: M = 131.4, 95% CI [95.2; 167.6] and Memory Lane: M = 155.8, 95% CI [123.5; 188.2]) compared to the sham group (i.e., Bulky Codes: M = 38.1, 95% CI [27.7; 48.5] and Memory Lane: M = 50.4, 95% CI [32.9; 67.8])."

17a) For each primary and secondary outcome, results for each group, and the estimated effect size and its precision (such as 95% confidence interval)

Does your paper address CONSORT subitem 17a? *

Copy and paste relevant sections from the manuscript (include quotes in quotation marks "like this" to indicate direct quotes from your manuscript), or elaborate on this item by providing additional information not in the ms, or briefly explain why the item is not applicable/relevant for your study

"Comparisons between the baseline and post-treatment assessments (see figure 2) showed significant differences between the two groups in the domain for pro-cessing speed and attention (P = .019) as well as the domain for working memory (P < .001). Closer inspection revealed that the differences were driven by higher mean change scores in the experimental group (processing speed and attention: M = 9.93, 95% CI [-61.2; 81.0] and working memory: M = 134.8, 95% CI [102.2; 167.5]) compared to the sham group (processing speed and attention: M = -72.8, 95% CI[-100.1; -45.6]; and working memory (M = 38.6, 95% CI [22.8; 54.4]). Re-garding the individual games in these domains, significant differences were only present for Bulky Codes (P < .001) and Memory Lane (P < .001), which were driv-en by improvements in the experimental group (i.e., Bulky Codes: M = 131.4, 95% CI [95.2; 167.6] and Memory Lane: M = 155.8, 95% CI [123.5; 188.2]) compared to the sham group (i.e., Bulky Codes: M = 38.1, 95% CI [27.7; 48.5] and Memory Lane: M = 50.4, 95% CI [32.9; 67.8])."

AND

"No significant differences between baseline, post-treatment, and 6-months follow-up assessments were observed between the two groups regarding DD, HDD, aver-age craving level, and highest craving level (see Figure 3). The mean difference from baseline to post-treatment assessment in alcohol consumption (DD: M = 0.87, 95% CI [-1.54; 3.27], P = 0.48; HDD: M = 0.85, 95% CI [-1.30; 3.01], P = .44) in favor of the sham group (M = -14.13, 95% CI [-16.67; -11.59]) rather than the experimental group (M = -13.27, 95% CI [-16.53; -10.00]). This was also evident for the mean craving level (MCL: 0.85, 95% CI [-0.69; 2.39], P = .28) which also showed a larger reduction in the sham group (M = -13.74, 95% CI [-16.32; -11.15]) rather than the experimental group (M = -12.88, 95% CI [-15.98; -9.78])."

17a-i) Presentation of process outcomes such as metrics of use and intensity of use

In addition to primary/secondary (clinical) outcomes, the presentation of process outcomes such as metrics of use and intensity of use (dose, exposure) and their operational definitions is critical. This does not only refer to metrics of attrition (13-b) (often a binary variable), but also to more continuous exposure metrics such as “average session length”. These must be accompanied by a technical description how a metric like a “session” is defined (e.g., timeout after idle time) [1] (report under item 6a).

	1	2	3	4	5	
subitem not at all important	○	○	○	○	◉	essential

Clear selection

Does your paper address subitem 17a-i?

Copy and paste relevant sections from the manuscript (include quotes in quotation marks "like this" to indicate direct quotes from your manuscript), or elaborate on this item by providing additional information not in the ms, or briefly explain why the item is not applicable/relevant for your study

"The first criterion specifying that more than half of the patients had to fulfill the minimum requirement of five completed game trials for each of the games was reached in five out of the six games in the experimental version of the Brain+ Alco-Recover application (see supplementary material, Table S1). The game that did not meet this criterion was Attention Island, where only 37.1% of the patients (n = 13) completed the five required game trials. In the sham version of the application, the criterion was reached in four out of the five games. The game that did not meet the criterion was Bulky Codes, where 48% of the patients completed the five required game trials. There was no significant difference between the two groups regarding the number of completed game trials, the only exception being that significantly more patients in the sham group completed the minimum number of required game trials for Attention Island (n = 13, 65.7%) compared to the experimental group (P = .043)."

17b) For binary outcomes, presentation of both absolute and relative effect sizes is recommended

Does your paper address CONSORT subitem 17b? *

Copy and paste relevant sections from the manuscript (include quotes in quotation marks "like this" to indicate direct quotes from your manuscript), or elaborate on this item by providing additional information not in the ms, or briefly explain why the item is not applicable/relevant for your study

We did not have binary outcomes in this study

18) Results of any other analyses performed, including subgroup analyses and adjusted analyses, distinguishing pre-specified from exploratory

Does your paper address CONSORT subitem 18? *

Copy and paste relevant sections from the manuscript (include quotes in quotation marks "like this" to indicate direct quotes from your manuscript), or elaborate on this item by providing additional information not in the ms, or briefly explain why the item is not applicable/relevant for your study

"Finally, the primary analyses for the acceptability outcome were followed up with secondary analyses to explore the number of patients who completed the re-quired training duration or the 150 games regardless of game type. The analyses showed that 31.4 % (n =11) and 33.4% (n =12) of the patients in the experimental group and sham group, respectively, reached this requirement (see supplementary material, Table S2, more information on the individual games)."

18-i) Subgroup analysis of comparing only users

A subgroup analysis of comparing only users is not uncommon in ehealth trials, but if done, it must be stressed that this is a self-selected sample and no longer an unbiased sample from a randomized trial (see 16-iii).

	1	2	3	4	5	
subitem not at all important	(x)	( )	( )	( )	( )	essential

Clear selection

Does your paper address subitem 18-i?

Copy and paste relevant sections from the manuscript (include quotes in quotation marks "like this" to indicate direct quotes from your manuscript), or elaborate on this item by providing additional information not in the ms, or briefly explain why the item is not applicable/relevant for your study

We used intention-to-treat analyses, and exploratory analyses, which were per-protocol

19) All important harms or unintended effects in each group
(for specific guidance see CONSORT for harms)

Does your paper address CONSORT subitem 19? *

Copy and paste relevant sections from the manuscript (include quotes in quotation marks "like this" to indicate direct quotes from your manuscript), or elaborate on this item by providing additional information not in the ms, or briefly explain why the item is not applicable/relevant for your study

This study was based on a publicly available training application, which was not deemed as harmful or posing any adverse effects.

19-i) Include privacy breaches, technical problems

Include privacy breaches, technical problems. This does not only include physical “harm” to participants, but also incidents such as perceived or real privacy breaches [1], technical problems, and other unexpected/unintended incidents. “Unintended effects” also includes unintended positive effects [2].

	1	2	3	4	5	
subitem not at all important	○	○	○	○	◉	essential

Clear selection

Does your paper address subitem 19-i?

Copy and paste relevant sections from the manuscript (include quotes in quotation marks "like this" to indicate direct quotes from your manuscript), or elaborate on this item by providing additional information not in the ms, or briefly explain why the item is not applicable/relevant for your study

We had technical issues with the app, which have been addressed in some of the earlier items. These included a bug that resulted in an assessment of only two cognitive domains. Furthermore, we could not log the actual duration spent in each game, so we were only able to look at the number of completed games.

19-ii) Include qualitative feedback from participants or observations from staff/researchers

Include qualitative feedback from participants or observations from staff/researchers, if available, on strengths and shortcomings of the application, especially if they point to unintended/unexpected effects or uses. This includes (if available) reasons for why people did or did not use the application as intended by the developers.

	1	2	3	4	5	
subitem not at all important	(•)	( )	( )	( )	( )	essential

Clear selection

Does your paper address subitem 19-ii?

Copy and paste relevant sections from the manuscript (include quotes in quotation marks "like this" to indicate direct quotes from your manuscript), or elaborate on this item by providing additional information not in the ms, or briefly explain why the item is not applicable/relevant for your study

We did not include qualitative feedback or observations, as this was not a part of the present trial. Instead our aim was only to assess any potential and preliminary effects of the application as well as its feasibility, before we could progress to a second trial.

DISCUSSION

22) Interpretation consistent with results, balancing benefits and harms, and considering other relevant evidence

NPT: In addition, take into account the choice of the comparator, lack of or partial blinding, and unequal expertise of care providers or centers in each group

22-i) Restate study questions and summarize the answers suggested by the data, starting with primary outcomes and process outcomes (use)

Restate study questions and summarize the answers suggested by the data, starting with primary outcomes and process outcomes (use).

	1	2	3	4	5	
subitem not at all important	○	○	○	○	◉	essential

Clear selection

Does your paper address subitem 22-i? *

Copy and paste relevant sections from the manuscript (include quotes in quotation marks "like this" to indicate direct quotes from your manuscript), or elaborate on this item by providing additional information not in the ms, or briefly explain why the item is not applicable/relevant for your study

"The present pilot and feasibility study provides the most recent evidence on the potential usability and effectiveness of an unguided, smartphone-based, serious gaming application administered as add-on to conventional treatment among pa-tients with AUD. The Brain+ Alco-Recover application was deemed feasible and ac-cepted by the patients. Further, the experimental group exhibited significant improvements in attention and working memory compared to the sham group. Despite these promising effects, no significant differences were found between the two groups regarding clinical outcomes. Nonetheless, the observed reduction in alcohol consumption in the experimental group suggests that this type of cognitive training could potentially improve long-term clinical outcomes."

22-ii) Highlight unanswered new questions, suggest future research

Highlight unanswered new questions, suggest future research.

	1	2	3	4	5	
subitem not at all important	○	○	○	○	●	essential

Clear selection

Does your paper address subitem 22-ii?

Copy and paste relevant sections from the manuscript (include quotes in quotation marks "like this" to indicate direct quotes from your manuscript), or elaborate on this item by providing additional information not in the ms, or briefly explain why the item is not applicable/relevant for your study

"Lastly, the sham group was not exposed to training that targeted verbal learning and episodic memory [...] This should be addressed in future versions of the Brain+ Alco-Recover application as well as any other cognitive training programs, since learning and memory have been implicated in alcohol-related disorders [...] where it has been shown that memory functioning is less susceptible to the spontaneous recovery occurring with continuing abstinence 4"

20) Trial limitations, addressing sources of potential bias, imprecision, and, if relevant, multiplicity of analyses

20-i) Typical limitations in ehealth trials

Typical limitations in ehealth trials: Participants in ehealth trials are rarely blinded. Ehealth trials often look at a multiplicity of outcomes, increasing risk for a Type I error. Discuss biases due to non-use of the intervention/usability issues, biases through informed consent procedures, unexpected events.

	1	2	3	4	5	
subitem not at all important	○	○	○	○	◉	essential

Clear selection

Does your paper address subitem 20-i? *

Copy and paste relevant sections from the manuscript (include quotes in quotation marks "like this" to indicate direct quotes from your manuscript), or elaborate on this item by providing additional information not in the ms, or briefly explain why the item is not applicable/relevant for your study

"The present study has some limitations. First, the sample size may not have been adequate to detect significant changes in clinical outcomes between the experi-mental and sham groups. Second, cognitive performance was assessed using the in-game, pre-assessment program, which was performed prior to initiating the training program and after completion of the three-months training program."

21) Generalisability (external validity, applicability) of the trial findings

NPT: External validity of the trial findings according to the intervention, comparators, patients, and care providers or centers involved in the trial

21-i) Generalizability to other populations

Generalizability to other populations: In particular, discuss generalizability to a general Internet population, outside of a RCT setting, and general patient population, including applicability of the study results for other organizations

	1	2	3	4	5	
subitem not at all important	○	○	○	○	◉	essential

Clear selection

Does your paper address subitem 21-i?

Copy and paste relevant sections from the manuscript (include quotes in quotation marks "like this" to indicate direct quotes from your manuscript), or elaborate on this item by providing additional information not in the ms, or briefly explain why the item is not applicable/relevant for your study

"The reliance on the in-game assessment of cognitive functions also precludes bold comparisons to the effects of other cognitive training programs that were assessed with standardized instruments, thus reducing the external validity of the present findings."

21-ii) Discuss if there were elements in the RCT that would be different in a routine application setting

Discuss if there were elements in the RCT that would be different in a routine application setting (e.g., prompts/reminders, more human involvement, training sessions or other co-interventions) and what impact the omission of these elements could have on use, adoption, or outcomes if the intervention is applied outside of a RCT setting.

	1	2	3	4	5	
subitem not at all important	○	○	○	○	◉	essential

Clear selection

Does your paper address subitem 21-ii?

Copy and paste relevant sections from the manuscript (include quotes in quotation marks "like this" to indicate direct quotes from your manuscript), or elaborate on this item by providing additional information not in the ms, or briefly explain why the item is not applicable/relevant for your study

"In addition, the development of the Brain+ Alco-Recover application was in its early stages, and as a result more complex features as well as adaptations of specif-ic games were omitted. The lack of a notification system made it more difficult to remind the patients about their training sessions, and implementing such a feature should be emphasized in unguided training programs, as this could increase the number of completed games."

OTHER INFORMATION

23) Registration number and name of trial registry

Does your paper address CONSORT subitem 23? *

Copy and paste relevant sections from the manuscript (include quotes in quotation marks "like this" to indicate direct quotes from your manuscript), or elaborate on this item by providing additional information not in the ms, or briefly explain why the item is not applicable/relevant for your study

Trial Registration: IRRID: RR2-10.3389/fpsyt.2021.727001

24) Where the full trial protocol can be accessed, if available

Does your paper address CONSORT subitem 24? *

Cite a Multimedia Appendix, other reference, or copy and paste relevant sections from the manuscript (include quotes in quotation marks "like this" to indicate direct quotes from your manuscript), or elaborate on this item by providing additional information not in the ms, or briefly explain why the item is not applicable/relevant for your study

"This study was not registered in a public trials registry, but a protocol for the trial was published prior to the inclusion of patients 40."

25) Sources of funding and other support (such as supply of drugs), role of funders

Does your paper address CONSORT subitem 25? *

Copy and paste relevant sections from the manuscript (include quotes in quotation marks "like this" to indicate direct quotes from your manuscript), or elaborate on this item by providing additional information not in the ms, or briefly explain why the item is not applicable/relevant for your study

"Financial funding for this pilot and feasibility trial was granted by the Psychi-atric Research Foundation, which is a part of the Region of Southern Denmark. No additional financial support was received for conducting the trial."

X27) Conflicts of Interest (not a CONSORT item)

X27-i) State the relation of the study team towards the system being evaluated

In addition to the usual declaration of interests (financial or otherwise), also state the relation of the study team towards the system being evaluated, i.e., state if the authors/evaluators are distinct from or identical with the developers/sponsors of the intervention.

	1	2	3	4	5	
subitem not at all important	◉	○	○	○	○	essential

Clear selection

Does your paper address subitem X27-i?

Copy and paste relevant sections from the manuscript (include quotes in quotation marks "like this" to indicate direct quotes from your manuscript), or elaborate on this item by providing additional information not in the ms, or briefly explain why the item is not applicable/relevant for your study

There are no conflicts of interest

About the CONSORT EHEALTH checklist

As a result of using this checklist, did you make changes in your manuscript? *

- ○ yes, major changes
- ◉ yes, minor changes
- ○ no

What were the most important changes you made as a result of using this checklist?

Changes to the primary outcomes, and why only two are reported instead of the four domains specified in the protocol, and also the information on trial registration.

How much time did you spend on going through the checklist INCLUDING making changes in your manuscript *

I spent more than two and half hours in total

As a result of using this checklist, do you think your manuscript has improved? *

- ○ yes
- ○ no
- ◉ Other: Only a slight improvement compared to the version prior to completin[illegible]

Would you like to become involved in the CONSORT EHEALTH group?

This would involve for example becoming involved in participating in a workshop and writing an "Explanation and Elaboration" document

- ○ yes
- ◉ no
- ○ Other:

Clear selection

Any other comments or questions on CONSORT EHEALTH

Your answer

STOP - Save this form as PDF before you click submit

To generate a record that you filled in this form, we recommend to generate a PDF of this page (on a Mac, simply select "print" and then select "print as PDF") before you submit it.

When you submit your (revised) paper to JMIR, please upload the PDF as supplementary file.

Don't worry if some text in the textboxes is cut off, as we still have the complete information in our database. Thank you!

Final step: Click submit !

Click submit so we have your answers in our database!

Submit Clear form

Never submit passwords through Google Forms.

This content is neither created nor endorsed by Google. Report Abuse - Terms of Service - Privacy Policy

Google Forms
